# Integrated Single-Step Terahertz Metasensing for Simultaneous Detection Based on Exosomal Membrane Proteins Enables Pathological Typing of Gastric Cancer

**DOI:** 10.34133/research.0625

**Published:** 2025-03-10

**Authors:** Qingzhe Jia, Zhaofu Ma, Yujia Wang, Mingjin Zhang, Guijun Zou, Bin Lan, Songyan Li, Zeqiu Lao, Wenbin Shen, Jing Lou, Yanan Jiao, Xiaohui Du

**Affiliations:** ^1^ Department of General Surgery, First Medical Center, Chinese PLA General Hospital, Beijing 100853, China.; ^2^ Innovation Laboratory of Terahertz Biophysics, National Innovation Institute of Defense Technology, Beijing 100071, China.; ^3^ Department of General Surgery, The 901^st^ Hospital of PLA, Hefei 230031, Anhui Province, China.; ^4^ Department of Traditional Chinese Medicine, First Medical Center, Chinese PLA General Hospital, Beijing 100853, China.; ^5^ Department of Critical Care Medicine in 83rd Group Army Hospital, Xinxiang City 453000, Henan Province, China.; ^6^ Emergency Department, Seventh Medical Center, Chinese PLA General Hospital, Beijing 100010, China.

## Abstract

Exosomes (Exos) are emerging as noninvasive biomarkers for diagnosis and progression monitoring of gastric cancer (GC). However, the heterogeneity discrimination and ultrasensitive quantification of Exos presents a considerable analytical challenge, thereby impeding severely their clinical application. Herein, we propose an integrated terahertz metasensing platform for the discrimination of Exos in distinct subtypes of GC in a single step—through the simultaneous evaluation of the category and richness level of Exos membrane proteins. Characterized by dual-sided independent sensing capabilities with enhanced sensitivity (169 and 325 GHz per refractive index unit, respectively), the metasensor functionalized with antibodies simultaneously reflects the content of 2 membrane proteins in the terahertz spectral response. Our approach concurrently completes accurate differentiation and precise quantification of GC-subtype Exos by integrating dual-sided sensing information in merely a single assay. The dual-sided sensing design enhances the reliability of detection results. Moreover, combined with the signal amplification of gold nanoparticles, the platform experimentally demonstrates a superior dynamic response to Exos concentrations spanning from 1 × 10^4^ to 1 × 10^8^ particles/ml, with the limit of detection being 1 × 10^4^ particles/ml. This work provides new insights into multisensing metasurface design and paves the way for precise and personalized cancer treatment through the specific sensing of Exos.

## Introduction

Gastric cancer (GC), a formidable malignancy seriously affecting public health, witnessed more than 968,000 new cases and 660,000 fatalities globally in 2022 [1]. GC exhibits a spectrum of pathological subtypes, each with a unique therapeutic approach and distinct clinical outcomes. Individualized medicine necessitates accurate pathology-subtype categorization of diseases and continuous assessment of treatment efficacy [2]. Conventional pathological techniques, including tissue biopsy and immunohistochemistry, are frequently invasive and necessitate the involvement of specialized personnel, entailing intricate procedures. These methods are challenging for cancer-subtype differentiation and real-time assessment of tumor status [3]. Different pathological subtypes of GC release distinct subsets of exosomes (Exos), which enables the detection of Exos to evaluate GC typing and tumor burden based on liquid biopsy with the merit of noninvasiveness and convenience [4]. Exos, nanoscale membrane vesicles, are widely present in the blood. These vesicles encapsulate a wealth of bioactive molecules, including proteins, nucleic acids, and lipids, which play a crucial role in intercellular communication [5–7]. However, Exos exhibit a high degree of heterogeneity, manifested as the vesicles contain multiple key membrane proteins with differences in expression levels [8]. Correspondingly, the heterogeneity of Exos derived from distinct GC subtypes is primarily characterized by variations in the content of key membrane proteins. Notably, cluster of differentiation 97 (CD97) and high mobility group box 1 (HMGB1) promote tumor cell metastasis and are crucially correlated with patient clinical stage and prognosis. The heterogeneity of membrane proteins makes it difficult to accurately distinguish Exos based on a single membrane protein [9]. Therefore, detecting multiple membrane proteins simultaneously to characterize Exos could potentially be a pivotal strategy for achieving subtype recognition of GC.

Terahertz (THz) waves, which reside in the frequency interval between the infrared and microwave bands, are increasingly recognized for their remarkable potential in the biomedical domain, featuring low photon energy, strong penetrability, and the fingerprint spectral response for various materials [10–16]. Despite these advantages, the THz detection method still faces considerable technical challenges. For example, it is extremely difficult to diagnose high-conductivity bulk materials due to very limited penetration depths for THz and other electromagnet waves. By using the novel method of optical-pump reflection-style THz-probe spectroscopy, the ultrafast dynamics of charge carrier performance was successfully measured for high-conductivity bulk substances [17]. Furthermore, the insufficiency of THz radiation power, in conjunction with the mismatch between the matter and the THz wavelength, leads to the limited sensitivity of THz biosensing techniques [18–21]. The advent of metasurfaces with field-localized capability is undoubtedly a solution to the bottleneck of THz technology in the biomedical field.

As artificial structures with subwavelength meta-atoms arranged periodically, metasurfaces showcase remarkable performance in manipulating the phase, amplitude, and polarization of electromagnetic waves [22–27]. Diverse resonant modes of metasurfaces, such as anapoles, Fano resonances, and electromagnetically induced transparency, can generate highly localized strong electric fields to maximize light–biomaterial interaction [28–34]. Among these, bound states in the continuum (BICs), which have the capability to tailor resonances with arbitrarily large quantity (*Q*) factors, are earning considerable attention [35,36]. Theoretically, BICs act as intrinsic wave states that trap light with infinite lifetimes, but their nonradiative property characterized by a vanishing spectral linewidth restricts their practical application [37,38]. Judicious symmetry breaking enables BICs to convert into quasi-BICs (QBICs) with finite radiation leakage, offering notable opportunities for the innovative design of high-*Q*-factor optical devices [39,40]. THz metasensing, assisted by immune functionalization technology, facilitates the detection of specific proteins, such as spike proteins and C-reactive protein, which prove advantageous in the diagnosis and assessment of various diseases. It is imperative within the individualized medicine realm to differentiate between various types of Exos, thus necessitating the creation of innovative multidimensional metasensing approaches to address the heterogeneity in membrane protein composition.

In this work, we introduce a dual-sided THz metasurface biosensor, simultaneously achieving the precise identification and ultrasensitive quantitation of GC-subtype Exos by detecting 2 membrane proteins in one step. The devices are characterized by independent sensing function based on 2 distinct QBICs with considerable sensitivity (169 and 325 GHz/RIU, respectively). Modifying the dual sides with 2 distinct antibodies (anti-CD97 and anti-HMGB1), we successfully accomplish precise capture of Exos expressing specific membrane protein antigens. Due to antigen–antibody specific binding, the number of captured Exos are determined by the composition of membrane proteins present in Exos. The spectral frequency shifts in QBIC I and QBIC II correspond to the content of the 2 membrane proteins CD97 and HMGB1, respectively. Comparing the frequency shifts of the 2 QBICs, our method effectively distinguished Exos from various GC subpopulations cell lines (MGC-803 and SGC-7901) and normal gastric cells line (GES-1) in a single step. The dual-sided biosensor enhances the reliability of detection results and shows an excellent dynamic response to trace detection concentrations of GC Exos in the range of 1 × 10^4^ to 1 × 10^8^ particles/ml, with a limit of detection (LOD) reaching 1 × 10^4^ particles/ml. Consequently, through an integrative analysis of the frequency shift data associated with the 2 QBICs, our biosensor simultaneously achieves both the precise discrimination and ultrasensitive quantitation of GC-subtype Exos in a single assay. Therefore, this work provides an efficient biosensing platform for accurate diagnosis and pathological typing of GC to guide precise individualized treatment.

## Results

### Operational mechanism of the dual-sensing biosensor for GC-Exos subtype recognition in a single step

**Fig. 1.**
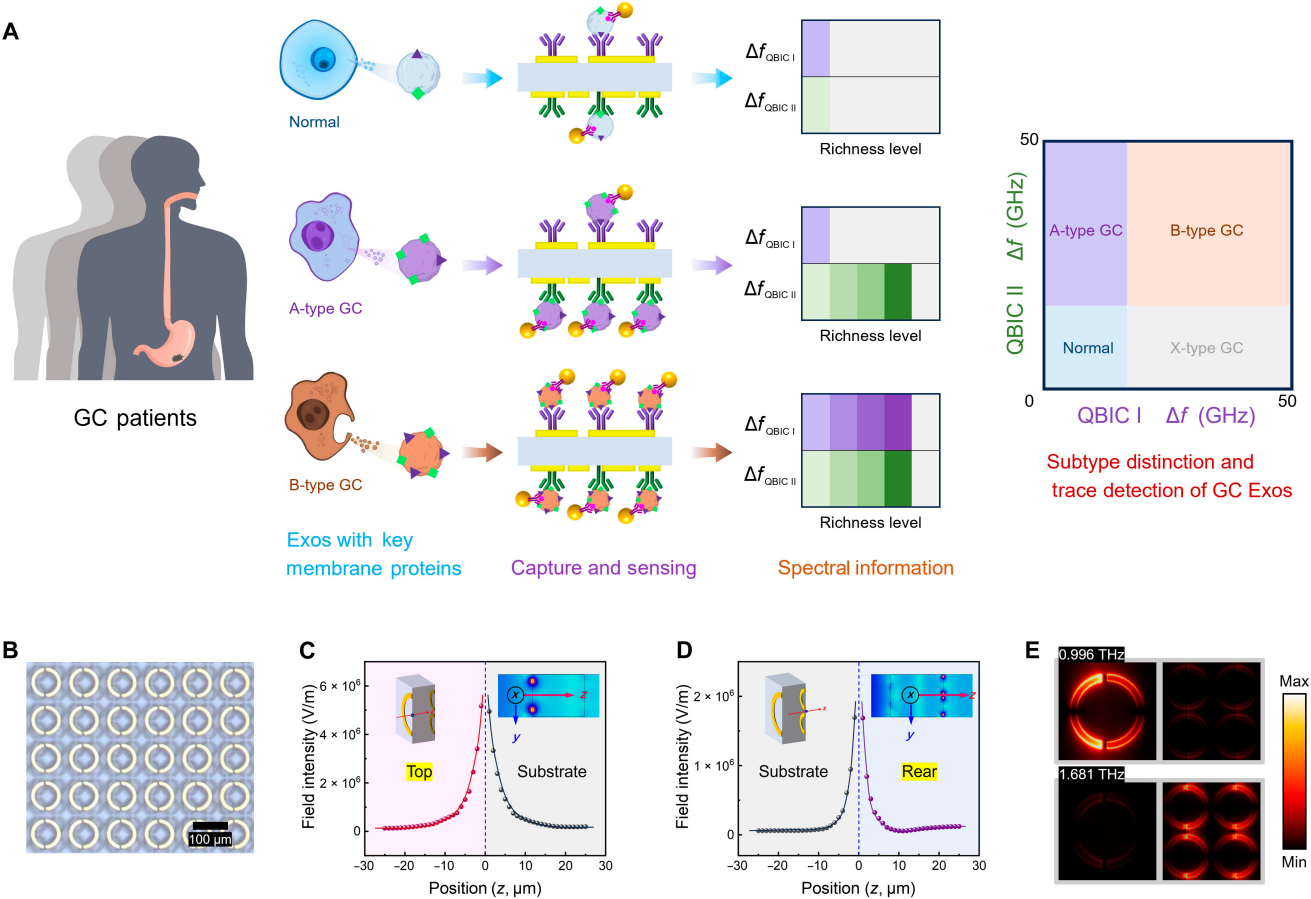
Schematic of the metasurface biosensor for gastric cancer (GC)-subtype exosome (Exos) differentiation in one step. (A) Personalized therapy relies on accurate categorization of tumors and continuous assessment in real time. Different pathological subtypes of GC indeed release distinct subsets of Exos with evident heterogeneity. Simultaneously evaluating the abundance of membrane proteins CD97 and high mobility group box 1 (HMGB1) is necessary to distinguish Exos subtypes. The metasurfaces with dual-sensing capacity are respectively functionalized with anti-CD97 and anti-HMGB1 to capture Exos modified with gold nanoparticles (AuNPs). The heterogeneity of membrane proteins dictates the variation in the Exos quantity that can be bound by specific antibodies. The Exos concurrently captured on the top and rear sides produce a corresponding frequency shift in quasi-bound state in the continuum (QBIC) I and QBIC II, which separately reflect the richness of CD97 and HMGB1. Based on the comprehensive analysis of the 2 QBICs, the biosensor is capable of discriminating Exos derived from various GC subtypes and can also ultrasensitively quantify exosomal concentrations in a single assay. (B) The optical micrograph of the prepared metasurface with 2 distinct layers of asymmetric split-ring resonators (ASRs), fabricated on a quartz substrate (scale bar, 100 μm). (C) The top and (D) rear sides’ spatial distribution of the fringing *E* field. The orientation of the red arrow is defined as the positive value along the *Z* axis; the excited resonant structure is defined at the zero point. (E) *E*-field distribution of dual-sided metasensor at different resonant frequencies of 0.996 and 1.681 THz. The *E*-field distribution in simulation confirms that the metasurface displays dual independent sensing modes, ensuring the synchronous detection of Exos membrane proteins mentioned above.

Figure 1 depicts the working principle of our biosensing approach, facilitating the concurrent assessment of Exos membrane proteins in a single procedure to delineate the pathological subcategories of GC. The heterogeneity of Exos derived from different subtypes of GC is mainly manifested by differences in the abundance of key membrane proteins (CD97 and HMGB1). Consequently, detecting a single specific membrane protein is insufficient for accurately characterizing the variations in type and concentration of Exos. The metasurfaces with dual-sided independent sensing capability are functionalized with anti-CD97 and anti-HMGB1 to specifically capture Exos with membrane protein heterogeneity. Exos to be tested are conjugated with gold nanoparticles (AuNPs), which serve as THz signal amplifiers to enhance the sensitivity of the detection. The existence of membrane proteins CD97 and HMGB1 facilitates the specific capture of Exos by the top and rear surfaces of the metasurface, respectively, resulting in frequency shifts in THz transmission responses. Based on highly selective capture of Exos through antigen–antibody binding, the content of exosomal membrane proteins is reflected in the frequency shift of THz resonance. Since the dual-sided biosensor exhibits 2 independent QBIC resonance peaks with enhanced sensitivity, the frequency shifts in the transmission spectra of QBIC I and QBIC II synchronously display the expression levels of the membrane proteins CD97 and HMGB1 in Exos. Finally, by comprehensive analysis of 2 QBICs to form frequency shift area coding, distinct-color regions correspond to various subtypes of GC-derived Exos. Meanwhile, the measured magnitude of the frequency alteration serves as a discernible indicator of the concentration of these Exos (Fig. 1A). Accordingly, the dual-sided THz metasensors concurrently attain precise identification and ultrasensitive quantitative detection of Exos subtypes in a single assay, holding considerable promise for the accurate diagnosis and pathological categorization of GC.

### The principle and performance of the dual-sided sensing

To address the heterogeneity of membrane proteins within Exos, a dual-sided independent sensing strategy is critical. Figure 1B presents an optical microscopy image of the fabricated metasurface. The structure parameters and actual photograph are shown in Fig. S1. The simulated and experimental transmission spectra as well as the *Q* factors of the designed metasurface are presented in Fig. S2. Aiming to substantiate the principle of dual-sided QBIC, we simulated the *E*-field intensity on both sides of the metasurface at the *Y*–*Z* cross-section, as depicted in Fig. 1C and D. The images clearly indicate that the *E*-field intensity on both sides exhibits an exponential decay with spatial range. When the spatial range exceeds 20 μm, the *E*-field strength approaches 0. The thickness of substrate is 100 μm, maintaining the independence and noncoupling of the dual sensing. Furthermore, the confinement of the *E*-field intensity situated within the *X*–*Y* axis plane around the metallic structure is simulated, as depicted in Fig. 1E. When the resonance frequency is at 0.996 THz, the top side with a strong *E* field intensity is excited by THz waves. Similarly, at a resonance frequency of 1.681 THz, the rear side, characterized by an enhanced *E*-field intensity, is effectively excited. The magnetic field intensity and surface current distribution across the metasurface in simulation are shown in Fig. S3. Therefore, the simulation results demonstrate that the dual-sided metasurface can be simultaneously excited by THz waves, achieving the functionality of dual-sided sensing uncoupled with each other. The sensing performance of the metasurface is explored by altering the refractive index (*n*) of the analyte covering the top or rear side, as shown in Fig. S4. The calculated sensitivities of QBIC I and QBIC II are 169 and 325 GHz/refractive index unit (RIU), respectively. The sensing performance of the designed dual-sided metasurfaces are comparable to that of previous single-sided QBIC metasensors, showing a broad application prospect in the domain of biological detection.

### Heterogeneity of membrane proteins in diverse Exos subtypes

**Fig. 2.**
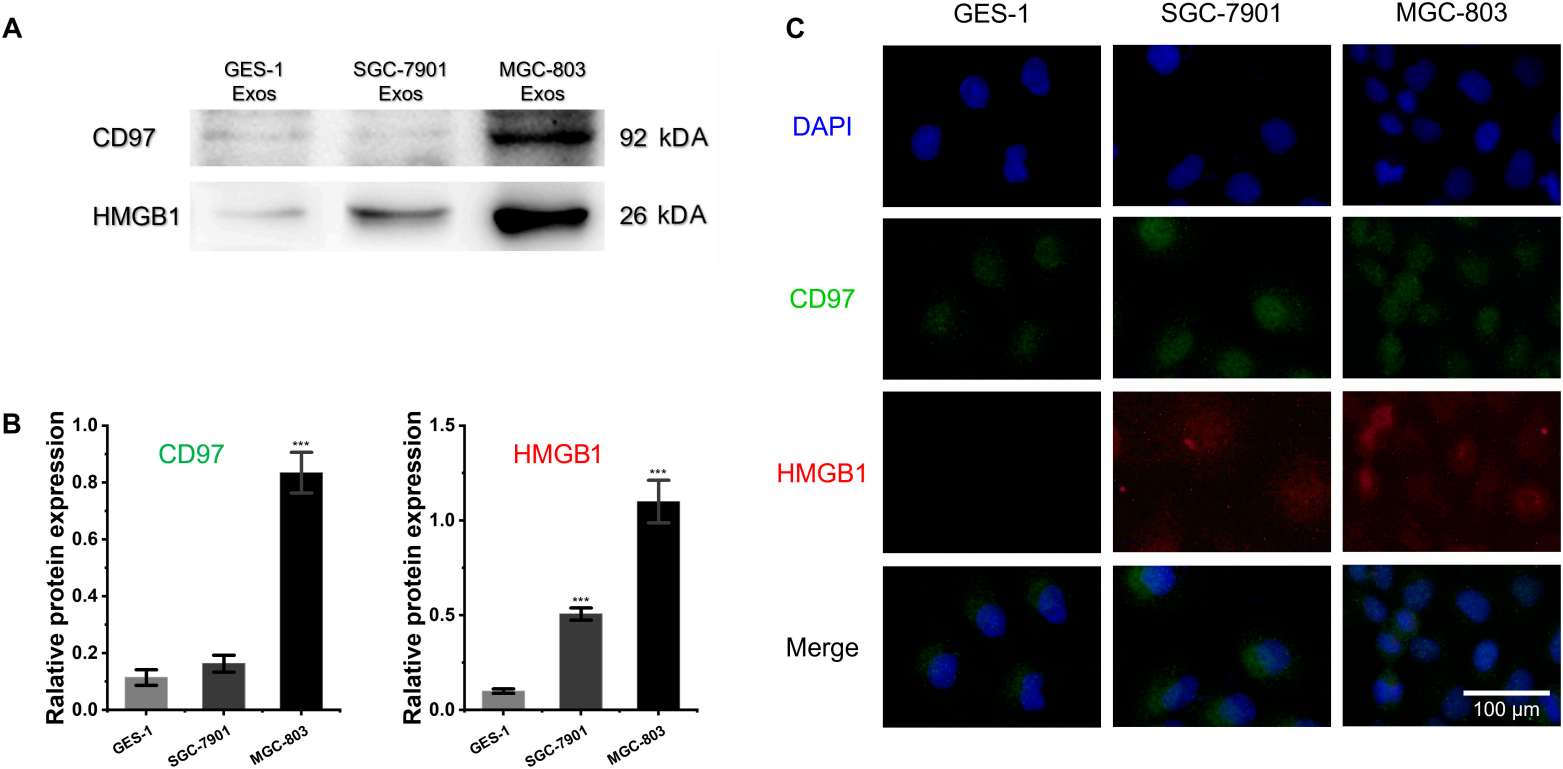
The protein expression heterogeneity corresponding to 3 Exos and their parent cells. (A) Western blot analysis of CD97 and HMGB1 in the Exos derived from MGC-803, SGC-7901, and GES-1. (B) Relative grayscale values of CD97 and HMGB1 in 3 types of Exos. The *P* values corresponding to “***”, from left to right, are 1.4 × 10^−4^, 8.6 × 10^−4^, and 7.1 × 10^−5^, respectively. (C) Fluorescence microscopy images corresponding to the localization and expression of CD97 and HMGB1 in 3 kinds of cells. DAPI, 4′,6-diamidino-2-phenylindole.

Membrane proteins play a vital role within Exos, serving as crucial elements that govern their functionality and mirror their distinctiveness. The Exos released by distinct subtypes of GC cells exhibit high heterogeneity, especially reflected in the types and richness level of Exos membrane proteins. The exosomal membrane protein HMGB1 is recognized for its role in enhancing the proliferative, migratory, and invasive capabilities of GC cells. CD97, a member of the epidermal growth factor family, is often overexpressed in GC. An elevated expression of CD97 is associated with GC cell dedifferentiation and aggressiveness and directly correlates with clinical pathological parameters, including tumor–node–metastasis classification. In order to validate the heterogeneity of Exos, Western blot detection was conducted to analyze the protein expression levels of CD97 and HMGB1 of 3 Exos derived from the GC cell lines MGC-803 and SGC-7901, as well as from the normal cell line GES-1, as shown in Fig. 2A. The grayscale values of the CD97 and HMGB1 in 3 Exos samples were normalized by comparison with the grayscale values of the internal reference protein (Fig. 2B). The differences of grayscale values are significant (*P* < 0.001). CD97 is relatively highly expressed in the Exos of MGC-803 and is almost not expressed in Exos from both the SGC-7901 and GES-1 cell lines. HMGB1 is highly expressed on the Exos derived from the MGC-803 cell line, moderately expressed in those from the SGC-7901 cell line, and virtually not expressed on Exos from GES-1 cells. Furthermore, the relative abundance and subcellular localization of the 2 proteins were analyzed using immunocytochemistry, which is shown in Fig. 2C. Both CD97 and HMGB1 are highly expressed in MGC-803 cells, while SGC-7901 cells contain only a moderate amount of HMGB1. Hence, given the unknown concentration of Exos and the heterogeneity of their protein richness, the concurrent detection of 2 membrane proteins as a strategy holds the potential for precise identification of different Exos subtypes.

### The functionalization of the dual-sided biosensor for capturing Exos concurrently

**Fig. 3.**
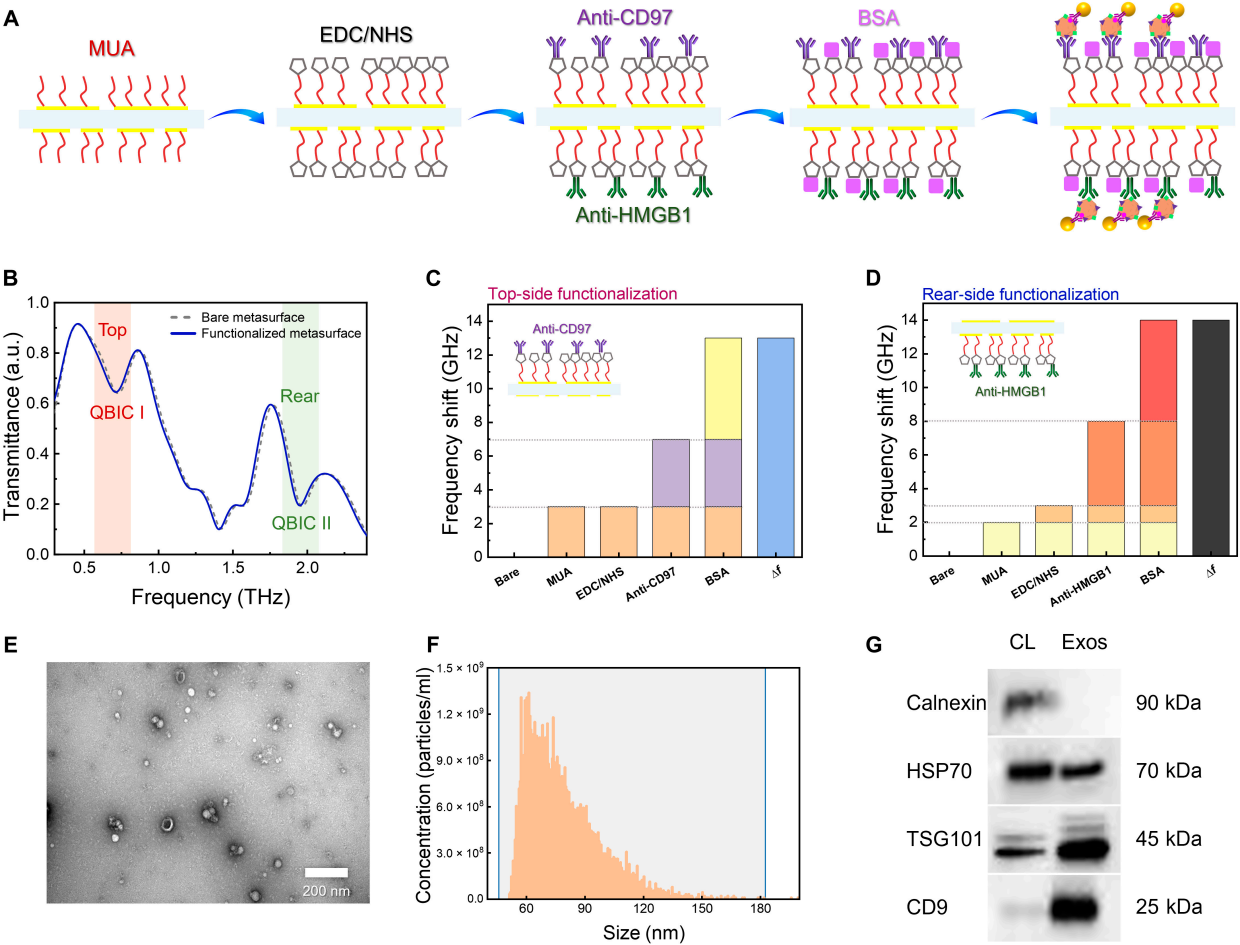
Functionalization of dual-sided metasurface and characterization of targeted Exos. (A) A schematic diagram of the steps associated with modifying 2 antibodies (anti-CD97 and anti-HMGB1) on the surface of a bifacial metasurface. (B) Experimental spectra of a dual-sided biosensor surface before and after functionalization. (C) The top and (D) rear sides’ spectral change for corresponding surface functionalization steps. (E) Transmission electron microscopy (TEM) images of Exos (scale bar, 200 nm). (F) The average size distribution of Exos. (G) Western blot detection of calnexin, heat shock protein 70 (HSP70), tumor susceptibility gene 101 protein (TSG101), and CD9 in the Exos derived from MGC-803. MUA, 11-mercaptoundecanoic acid; EDC, 1-ethyl-3-(3-(dimethylamino)-propyl) carbodiimide hydrochloride; NHS, *N*-hydroxysuccinimide; BSA, bovine serum albumin; CL, cell control.

The dual-sided biosensors are functionalized with anti-CD97 and anti-HMGB1 on each side, respectively, for subsequent detection. The detailed functionalization steps and the diagrammatic sketch of the detection method are shown in Fig. 3A. Initially, the metasurfaces are vertically immersed in 1 mM ethanolic solution of 11-mercaptoundecanoic acid (MUA) for over 12 h at 4 °C, which facilitates the self-assembly monolayer of MUA on both sides of the metasurfaces. Subsequently, the metasurfaces are thoroughly rinsed with deionized water to remove any residual MUA. Following this, the metasurfaces are vertically dipped in a mixture containing 400 mM 1-ethyl-3-(3-(dimethylamino)-propyl) carbodiimide hydrochloride (EDC) and 100 mM *N*-hydroxysuccinimide at 4 °C for 1 h, ensuring that the self-assembly monolayer is effectively activated for subsequent steps. Next, the metasurfaces are thoroughly immobilized by dipping in 5 ml of anti-CD97 and anti-HMGB1 solutions of 50 μg/ml, respectively. We utilize incubation dishes that consist of 2 rooms, sealed with dust-free adhesive tape to close the gaps, in order to functionalize both sides with different antibodies. Each metasurface is then washed with deionized water and dipped in a 10-ml bovine serum albumin solution of 50 μg/ml to block any free active sites on the surface. The unbound bovine serum albumin molecules are removed by deionized water, and the biosensors are ready for Exos detection. After the surface functionalization, the transmission spectral response of the biosensor experiences minor redshift (Fig. 3B), with the corresponding frequency shift of QBIC I and QBIC II being 13 GHz (Fig. 3C) and 14 GHz (Fig. 3D), respectively, which are considered as the baseline for detecting targeted Exos. Notably, the sensing platform exhibits a remarkable cost-effectiveness advantage, attributed to its exceptional reusability, as depicted in Fig. S5.

### The purification and characterization of Exos

The Exos from 3 different cell lines were extracted (see the “Exos isolation and purification” section) and the Exos derived from MGC-803 were experimentally characterized. Their morphology was characterized using transmission electron microscopy, revealing a distinct cup-shaped structure, as illustrated in Fig. 3E. The average size distribution of the Exos, centered at 75 nm, was ascertained through nanoparticle tracking analysis, as shown in Fig. 3F. Additionally, the presence of specific protein markers within the Exos lysates, including calnexin, heat shock protein 70, tumor susceptibility gene 101 protein, and CD9, was validated by Western blot, as detailed in Fig. 3G.

### Anti-CD9-AuNPs coupled to Exos as THz signal amplifiers

The synthesis steps for AuNPs conjugated with anti-CD9 can be found in the “Immunofunctionalization of AuNPs with anti-CD9” section. The Exos intended for detection were diluted to various concentration ranges, specifically from 1 × 10^4^ to 1 × 10^8^ particles/ml. These Exos at different concentrations were then combined with functionalized anti-CD9–AuNPs solution at a concentration of 1 × 10^11^ particles/ml and incubated in a shaking incubator for 1 h to promote the formation of Exos–anti-CD9–AuNPs complexes. The amplification effect of THz signals enhanced by AuNPs is illustrated in Fig. S6. In the experiment, we vertically immersed the dual-sided functionalized biosensors into a solution of Exos–anti-CD9–AuNPs at a specific concentration and incubated them for 30 min. Subsequently, the metasurfaces were vertically placed in deionized water and rinsed 5 times with shaking to remove unbound Exos. Then, blotting paper was used to absorb residual moisture, and the sample was allowed to dry at ambient temperature (26 ± 1 °C) for 6 min to prevent excess moisture from interfering with the THz signal. All measurements were performed at room temperature (26 ± 1 °C) and in a controlled dry environment with a humidity level maintained at 2% to minimize the influence of water vapor.

### Single-sensing detections fail to discriminate GC-subtype Exos

**Fig. 4.**
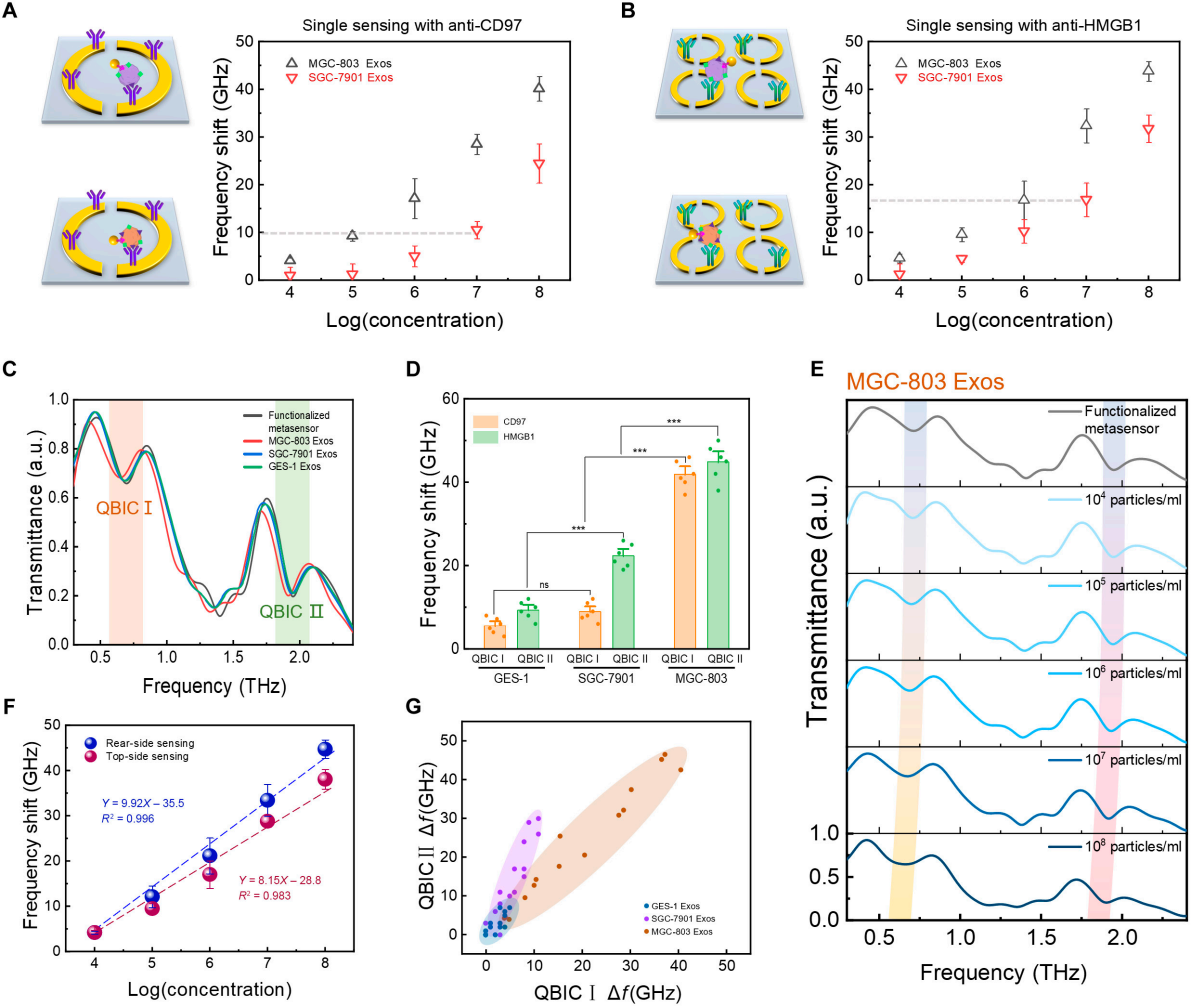
Dual-sensing strategy for precise differentiation and highly sensitive analysis of Exos. (A) Single-sided metasurface functionalized with CD97 for detecting GC-subpopulation Exos. (B) Single-sided metasurface functionalized with HMGB1 for detecting GC-subpopulation Exos. (C) The dual-sensing spectral response related to Exos from MGC-803, SGC-7901, and GES-1. (D) The frequency shifts of QBIC I and QBIC II corresponding to the 3 types of Exos. The *P* values corresponding to “ns” and “***”, from left to right, are 3.8 × 10^−1^, 1.1 × 10^−4^, 1.7 × 10^−8^, and 1.2 × 10^−6^, respectively. (E) The typical transmission spectra depending on MGC-803 Exos concentration from 1 × 10^4^ to 1 × 10^8^ particles/ml. (F) Fitting curves of the top-side and rear-side frequency shifts. (G) Extracted spectral frequency shifts of QBIC I and QBIC II corresponding to Exos with varying concentrations from 1 × 10^4^ to 1 × 10^8^ particles/ml that are derived from 2 GC cells and 1 normal cell. Distinct color-coded regions delineate specific Exos derived from various GC subtypes, and the extent of the frequency shift is indicative of the concentration of Exos.

Two single-sided metasurfaces based on the parameters of the dual-sided metasurface were manufactured and subjected to immune functionalization with anti-CD97 and anti-HMGB1, respectively. We employed metasurfaces functionalized with anti-CD97 to detect Exos derived from the MGC-803 and SGC-7901 within a concentration range of 1 × 10^4^ to 1 × 10^8^ particles/ml. As depicted in Fig. 4A, both types of Exos elicit increasingly substantial frequency shifts in response to rising concentrations. In the context of Exos with unknown concentrations and unidentified subtypes, the frequency shift induced by a single-side biosensor in one step, such as a 10-GHz shift, may be indicative of either MGC-803 Exos at a concentration of 1 × 10^5^ particles/ml or SGC-7901 Exos at a concentration of 1 × 10^7^ particles/ml. Notably, both MGC-803 and SGC-7901 Exos contain the CD97 protein, which complicates the differentiation based on single-protein detection. Although SGC-7901 Exos have a lower CD97 content compared to MGC-803, higher concentrations of SGC-7901 Exos can induce equivalent frequency shifts. As shown in Fig. 4B, after functionalizing another metasurface with anti-HMGB1, the same detection results can be observed. Therefore, given the intricate nature of Exos heterogeneity, it is evident that depending exclusively on the recognition of a solitary membrane protein, using a unilateral sensing approach is inadequate for the trustworthy differentiation of Exos subcategories.

### The dual-sided biosensor enables the simultaneous discrimination and quantification of Exos subtypes in a single step

We next implemented the dual-sided biosensor to detect Exos derived from different GC cell lines (MGC-803 and SGC-7901) and normal GES-1. Exos (1 × 10^8^ particles/ml) derived from 3 cell lines were experimentally measured separately. The measured transmission spectra of these Exos are depicted in Fig. 4C. When MGC-803-derived Exos were present, both QBIC I and QBIC II exhibited notable frequency shifts, specifically 38.4 and 44.2 GHz, respectively. SGC-7901-derived Exos induced a frequency shift of 11.3 GHz for QBIC I and a relatively more noticeable shift of 23.7 GHz for QBIC II. In contrast, Exos from GES-1 resulted in minimal frequency shifts at both QBIC I and QBIC II, with 6.8 and 10.2 GHz, respectively. We carried out the experiment with 6 measurements, and the frequency shifts corresponding to the 3 types of Exos are depicted in Fig. 4D. The differences in frequency shift are significant (*P* < 0.001). These investigations demonstrated that the dual-sided biosensors selectively captured Exos adorned with the intended proteins and effectively discriminated Exos derived from various subtypes of GC cells and normal cells. Therefore, the result indicates that our dual-sided biosensor successfully distinguished between the 3 types of Exos. It not only differentiated between Exos from cancerous and normal cells but also identified specific Exos from subpopulations of GC cells.

To further demonstrate the ultrasensitive detection capability of our dual-sided biosensor, we investigated its sensitivity in an assay quantitatively detecting Exos. Figure 4E illustrates the typical transmission spectra that are dependent on MGC-803 Exos concentration, along with a wide dynamic response range extending from 1 × 10^4^ to 1 × 10^8^ particles/ml. As the concentration of Exos progressively rises, the functionalized metasurface captures Exos via antibody–antigen binding, and the corresponding spectra of QBIC I and QBIC II demonstrate a regular redshift. We performed 3 measurements to test the stability of the sensing results. The frequency shift change (Δf) exhibits a linear relationship with the logarithm of Exos concentration ($C_{\text{Exos}}$) across 5 orders of magnitude (Fig. 4F), with the regression equation of the top side expressed as $\Delta f=8.15\mathrm{lg}\;C_{\mathrm{Exos}}-28.8$ $\left(R^{2}=0.983\right)$ and the regression equation of the rear side expressed as $\Delta f=9.92\mathrm{lg}\;C_{\text{Exos}}-35.5$ $\left(R^{2}=0.996\right)$. The LOD for the dual-sided biosensor was 1 × 10^4^ particles/ml. These results indicate that our dual-sided biosensors exhibit relatively stable detection capabilities across a wide range of concentration gradients, which can markedly enhance the detection accuracy. We additionally carried out spectroscopic analysis on Exos originated from SGC-7901 and GES-1 cells with varying concentrations from 1 × 10^4^ to 1 × 10^8^ particles/ml (Fig. S7). The spectral responses of Exos derived from diverse cells and at varying concentrations were extracted. The frequency shifts of QBIC I and QBIC II corresponding to these Exos are displayed in Fig. 4G. The blue area corresponds to the Exos derived from normal gastric cells GES-1, the purple area corresponds to the Exos derived from GC cells SGC-7901, and the yellow area corresponds to the Exos derived from another GC cells MGC-803. Furthermore, a robust linear correlation is observed between the frequency shifts and the concentration of GC-subtype Exos. Clearly, when the concentration of Exos exceeds 1 × 10^5^ particles/ml, even if the exact concentration is unknown, the biosensor with dual-sided sensing function can concurrently accomplish qualitative identification and quantitative detection of Exos in merely one step, which constitutes an aspect that prior single-sensing metasurfaces were incompetent to attain [37,40].

The developed sensing platform was utilized to analyze Exos samples from patients with GC and healthy controls. Further details are presented in Table S1 and Figs. S8 to S10. The platform exhibited preliminary potential for differentiating various clinical pathological types of GC. As shown in Table S2, compared with existing diagnostic methods [41–44], our biosensor introduces a novel dual-sensing paradigm, demonstrating a broad dynamic response range and an ultralow detection sensitivity (1 × 10^4^ particles/ml). The antigen–antibody binding reaction between the captured antibody and the Exos membrane proteins ensures high detection specificity. The sensing strategy we propose eliminates the need for complex sample pretreatment, thereby significantly enhancing detection efficiency with an approximate detection time of 1.5 h. The outcomes of our dual-sided detection system can be mutually verified, which undoubtedly enhances the precision and reliability of trace detection. Experimental evidence indicates that our dual-sided sensing strategy effectively addresses the challenge of Exos heterogeneity and is capable of discerning GC-subtype Exos, underscoring its marked potential in the precise diagnosis and tailored treatment of GC.

## Discussion

Various subtypes of GC cells release diverse heterogenous Exos. Utilizing the liquid biopsy technique to identify these vesicle subtypes makes it feasible to profile different types of GC, thereby introducing a fresh approach for precise diagnosis and tailored treatment of this malignancy. We experimentally demonstrated that 2 key membrane proteins, CD97 and HMGB1, are differentially expressed on GC-subtype Exos. The typical heterogeneity and inexact concentration of clinical samples to be tested make it difficult to identify Exos subtypes relying on the single-sensing strategy. To concurrently evaluate the content of 2 prominent membrane proteins, we propose a dual-sided sensing strategy based on dual QBIC with elevated sensitivity. Functionalized with distinct antibodies related to Exos-specific membrane proteins, the metasurface simultaneously detects 2 Exos membrane proteins in a single assay. In the trace detection of Exos, both sides of the biosensor show a large concentration response range, increasing the detection accuracy. Moreover, by modifying the captured antibodies on the metasurface, our sensing approach may enable the detection of other key exosomal membrane proteins, rendering it suitable for the diagnosis and personalized treatment of tumors, including breast cancer and pancreatic cancer. This demonstrates the extensive scalability and considerable application potential of our sensing strategy.

In summary, we theoretically and experimentally illustrated a novel THz metasensor with dual-sided sensing capabilities, based on synchronously assessing 2 pivotal membrane proteins, clinically accomplishing the discrimination of GC-subtype Exos in a single step. The dual-sided QBIC resonances with enhanced sensitivity (169 and 325 GHz/RIU, respectively) are independent of each other without mutual interference. The biosensor is equipped with anti-CD97 and anti-HMGB1 on its top and rear sides, therefore accurately capturing Exos with specific membrane protein antigens. By comprehensive analysis of the dual BIC frequency shifts, we not only completed the distinction between GC-subtype Exos but also concurrently differentiated the concentration of Exos. Moreover, the designed dual-sided biosensor shows an excellent dynamic response to trace detection concentrations of GC Exos in the range of 1 × 10^4^ to 1 × 10^8^ particles/ml, with an LOD of 1 × 10^4^ particles/ml. Therefore, our biosensing strategy paves new avenues for the simultaneous precise recognition and quantitative detection of GC-subtype Exos, providing valuable insights for accurate diagnostic and personalized medicine in the field of GC research.

## Materials and Methods

### Cell lines

MGC-803, SGC-7901 (human GC cell lines), and GES-1 (human normal gastric epithelial cell line) were all purchased from the American Type Culture Collection. MGC-803, SGC-7901 were cultured with advanced Dulbecco’s modified Eagle medium with addition of 2% fetal bovine serum (FBS) and 1% penicillin and streptomycin. GES-1 was cultured with 1640 medium, supplemented with 10% FBS and 1% penicillin and streptomycin. Exos-depleted FBS was used instead of regular FBS for Exos production.

### Exos isolation and purification

Exos were isolated from the culture supernatants of 3 distinct cell lines using a sequential differential centrifugation protocol. Initially, the media were centrifuged at 300 g for 10 min to remove large cellular debris, followed by centrifugation at 2,000 g for 10 min to sediment possible apoptotic bodies and smaller debris. The final step involved centrifugation at 10,000 g for 30 min to pellet any residual cell fragments or biomolecule aggregates. After resuspension in phosphate-buffered saline, it was centrifuged again in the same manner to obtain Exos precipitation. The purified Exos were resuspended in phosphate-buffered saline for further analysis.

### Immunofunctionalization of AuNPs with anti-CD9

Before initiating the experimental procedures, the concentration of anti-CD9 was ascertained utilizing BCA Protein Assay Kits. Subsequently, the antibodies underwent purification via a desalting column and filtration. AuNPs with a 40-nm diameter were primed for activation by vigorous vortexing and subsequent incubation with a mixture of EDC and *N*-hydroxysuccinimide for 30 min. The supernatant was then carefully aspirated, and the activated AuNPs were resuspended in 1 ml of potassium phosphate buffer; 50 μg of anti-CD9 was introduced to the AuNP solution and incubated for 60 min to facilitate antibody conjugation. The reaction was terminated by the addition of hydroxylamine. The conjugated AuNPs were subjected to centrifugation at 3,800 RCF for 10 min to pellet the particles, and the supernatant was discarded. The pellet was then resuspended in NCX conjugate diluent and stored at a temperature of 4 °C.

### THz spectroscopy system measurements

The spectral data presented in this study were obtained utilizing a THz time-domain spectroscopy system (THz-TDS, TAS7500TS), procured from ADVANTEST Corporation, Japan. The TDS system was enclosed within a sealed acrylic chamber and integrated with a drying unit to regulate the humidity necessary for detection.

## Supplementary Material

20250310-1

## Data Availability

The data that support the findings of this study are available from the corresponding authors upon reasonable request.

## Supplementary Materials

Supplementary 1 Notes S1 to S9 Figs. S1 to S10 Tables S1 and S2
