# Erratum to “Integrated Single-Step Terahertz Metasensing for Simultaneous Detection Based on Exosomal Membrane Proteins Enables Pathological Typing of Gastric Cancer”

**DOI:** 10.34133/research.1247

**Published:** 2026-04-20

**Authors:** Qingzhe Jia, Zhaofu Ma, Yujia Wang, Mingjin Zhang, Guijun Zou, Bin Lan, Songyan Li, Zeqiu Lao, Wenbin Shen, Jing Lou, Yanan Jiao, Xiaohui Du

**Affiliations:** ^1^ Department of General Surgery, First Medical Center, Chinese PLA General Hospital, Beijing 100853, China.; ^2^ Innovation Laboratory of Terahertz Biophysics, National Innovation Institute of Defense Technology, Beijing 100071, China.; ^3^ Department of General Surgery, The 901^st^ Hospital of PLA, Hefei 230031, Anhui Province, China.; ^4^ Department of Traditional Chinese Medicine, First Medical Center, Chinese PLA General Hospital, Beijing 100853, China.; ^5^ Department of Critical Care Medicine in 83rd Group Army Hospital, Xinxiang City 453000, Henan Province, China.; ^6^ Emergency Department, Seventh Medical Center, Chinese PLA General Hospital, Beijing 100010, China.

In the Research Article “Integrated Single-Step Terahertz Metasensing for Simultaneous Detection Based on Exosomal Membrane Proteins Enables Pathological Typing of Gastric Cancer,” the authors have identified an inadvertent image misplacement in the Supplementary Materials [1].

In Supplementary Figure S8, due to a layout error, the image for sample “N1” was mistakenly placed as the image for sample “P7,” resulting in the duplicate appearance of the “P7” sample image in that figure. All authors have thoroughly reviewed the data and confirm that this error is purely presentational; it does not affect any data analysis, interpretations, or scientific conclusions reported in the main text.

The corrected Figure S8 has now been updated in the Supplementary Materials package, and the authors apologize for the oversight.

## Supplementary Materials

Supplementary 1 Supplementary Notes S1 to S9 Figs. S1 to S10 Tables S1 and S2
