# Supplementary material for: Integrated Single-Step Terahertz Metasensing for Simultaneous Detection Based on Exosomal Membrane Proteins Enables Pathological Typing of Gastric Cancer
Source: Research (Wash D C). 2025 Mar 10;8:0625. doi: 10.34133/research.0625 (PMC11891340; doi:10.34133/research.0625)
Supplement: Supplementary 1 — Notes S1 to S9 Figs. S1 to S10 Tables S1 and S2 [file research.0625.f1.zip › Revised-Supplementary information-R1-clean.docx]

**Integrated Single-step Terahertz Metasensing for Simultaneous Detection Based on Exosomal Membrane Proteins Enables Pathological Typing of Gastric Cancer**

Qingzhe Jia1,2,4†, Zhaofu Ma1,2†, Yujia Wang3†, Mingjin Zhang4, Guijun Zou1, Bin Lan1, Songyan Li1, Zeqiu Lao1, Wenbin Shen5, Jing Lou2*, Yanan Jiao1,6*, and Xiaohui Du1*

1Department of General Surgery, First Medical Center, Chinese PLA General Hospital, Beijing 100853, China.

2Innovation Laboratory of Terahertz Biophysics, National Innovation Institute of Defense Technology, Beijing 100071, China.

3Department of Traditional Chinese Medicine, First Medical Center, Chinese PLA General Hospital, Beijing 100853, China.

4Department of General Surgery, The 901st Hospital of PLA, Hefei 230031, Anhui Province, China.

5Department of Critical Care Medicine in 83rd Group Army Hospital, Xinxiang City 453000, Henan Province, China.

6Emergency Department, Seventh Medical Center, Chinese PLA General Hospital, Beijing 100010, China.

*Address correspondence to: [duxiaohui301@sina.com](mailto:duxiaohui301@sina.com) (X. D.); [loujing9486@163.com](mailto:loujing9486@163.com) (J. L.); [jiaoyanan301@163.com](mailto:jiaoyanan301@163.com) (Y. J.)

- **Supplementary Note 1**

The metasurfaces were manufactured by standard micro/nanomachining processes. First, a 100 µm-thick quartz substrate was treated with an acetone solution, sonicated, rinsed with deionized water, and dried with nitrogen gas. Then, the Top side of substrate was patterned using photolithographic technique, followed by the deposition of a 100 nm-thick gold (Au) film and a 10 nm-thick titanium (Ti) adhesion layer onto the substrate via magnetron sputtering. The Ti layer acts as an intermediary, improving the interface between the gold film and the substrate, and thus ensuring a more secure and long-lasting attachment. Subsequently, deep silicon etching was applied to remove the surplus material, leaving behind an array of the designed microstructure. The Rear side of the metasurface was fabricated in the same manner. Fig.S1A reveals the diagrammatic sketch of the prepared metasurface. The metasurface is meticulously structured with two distinct layers of asymmetric split-ring resonators (ASR). For clarity, we designate these two sides as the Top side and Rear side, respectively. The detailed structural design of the dual-sided metasurface is showed in Fig. S1B. The unit meta-atom on the Top side is composed of a semicircle with a diameter of *r*130 µm, width *w*15 µm, and an arc segment positioned at a distance *d*15 µm from the *Y*-axis. Each unit cell is a square periodically arranged along the *X*-axis and *Y*-axis with a period *P*180 µm. The unit meta-atom on the Rear side is proportionately reduced to almost half the size of the one on the Top side, with corresponding parameters labeled as *r*217.5 µm, *w*23 µm, *d*23 µm, and *p*240 µm, respectively. Quartz with a thickness *t*100 µm as the substrate. Fig.S1C is the physical image of the manufactured metasurface. The substrate thickness of the metasurface is 100 µm, as depicted in Fig. S1D.

**Fig. S1.** The structure parameters and actual photograph of the fabricated metasurface. (A) The graphic image of the metasurface. (B) Top side’ and Rear side’ geometrical configuration of the meta-atom. (C) The dimensions of the fabricated metasurfaces. (D) The substrate thickness of the metasurface is 100 µm.

- **Supplementary Note 2**

To further illustrate the mechanisms and the spectral response behind the excitation of the metasurface, numerical simulations were executed by employing the Computer Simulation Technology (CST) Microwave Studio 2022. Periodic boundary conditions and the perfectly matched layer (PML) absorbing boundary condition were employed to enhance the accuracy of the simulation. 20 meshes with tetrahedral subdivision were covered by one wavelength. The dielectric constant of the quartz substrate was 3.9 and its thickness was modeled as 100 μm. The electrical conductivity of Au and Ti was specified as 4.56×107, 1.8×107, respectively. The polarized incident THz wave was directed along the *Z*-axis, with its *E*-field oriented parallel to the *X*-axis. The theoretical transmission spectrum and corresponding *Q*-factors of the proposed metasurface with dual-sided independent sensing capabilities are illustrated in Fig.S2A and Fig. S2B. The THz transmission spectra of the fabricated metasensor and their corresponding *Q*-factors are shown in Fig.S2C and Fig.S2D. The corresponding simulated and experimental resonant frequencies are 0.996 THz and 1.681 THz, 0.726 THz and 1.961 THz, respectively. The simulated and experimental transmission spectra exhibit a high degree of consistency. In experimental measurements, the *Q*-factors of the fabricated metasurface's dual-QBICs are 6.8 and 14.3, respectively, which are lower than the theoretical values. This discrepancy arises because the theoretical model typically assumes an idealized structure, whereas the actual fabrication process introduces various imperfections, such as dimensional deviations and surface roughness. Furthermore, real materials exhibit inherent losses, including dielectric losses in the substrate and ohmic losses in the metallic components.


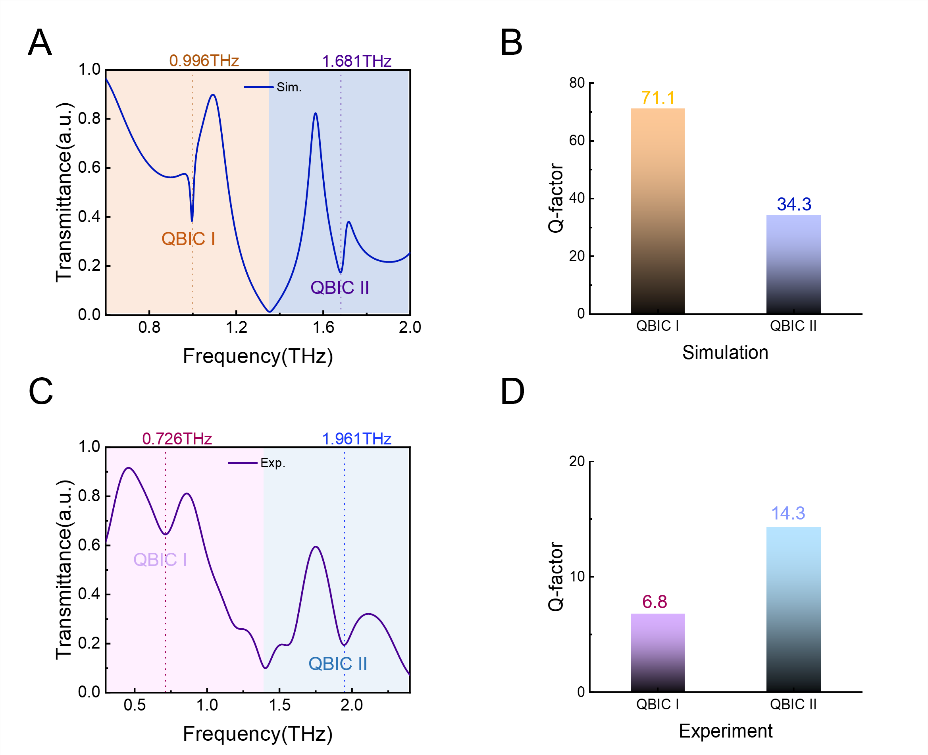


**Fig. S2.** The simulated and experimental transmission spectra as well as *Q*-factors of the designed metasurface. (A) The simulated transmission spectrum of the designed metasurface with resonant frequencies being 0.996 THz and 1.681 THz. (B) The *Q*-factors of the sensor in simulation. (C) The experimental transmission spectrum of the designed metasurface with resonant frequencies being 0.726 THz and 1.961 THz. (D) The *Q*-factors of the sensor in experiment.

- **Supplementary Note 3**

We performed digital simulations to probe the magnetic field intensity and surface current distributions across the metasurface, as shown in Fig.S3. Figure S3A illustrates that when tuned to the Top resonance frequency of 0.996 THz, the Top structure exhibits a markedly stronger magnetic field intensity compared to another side. Conversely, the Rear resonance at 1.681 THz demonstrates a significantly higher magnetic field intensity. Similarly, the surface current distributions depicted in Fig. S3B demonstrate that upon excitation of the resonant structure, a substantial surface current is induced on the corresponding unit cell, while the current on the opposite side of the structure is comparatively weaker.


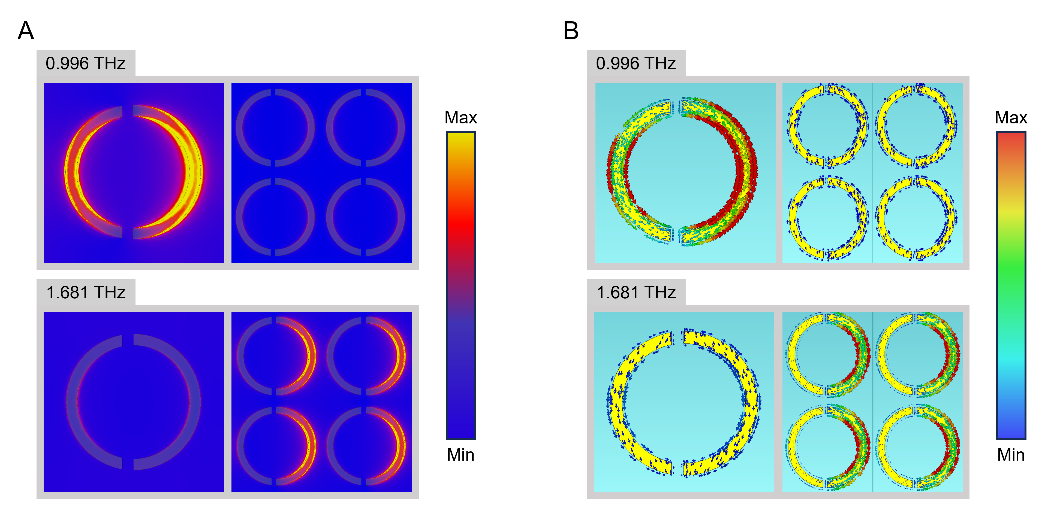


**Fig. S3.** The simulated magnetic field confinements and surface current distributions at two sides. (A) Magnetic field distribution when the Top and Rear sides are separately excited. (B) Surface current distributions when the Top and Rear sides are separately excited.

- **Supplementary Note 4**

To further explore the dual-sided sensing characteristics of the metasurface, we initially numerically investigated the metasurface's transmission spectra by altering the refractive index (*n*) of the analyte covering the Top or Rear side. QBIC I and QBIC II were designated to represent the QBIC excited on the Top and Rear side, respectively. In the simulation, the analyte was regarded as homogeneous dielectric with a thickness of 5 µm. As shown in Fig. S4A, when the refractive index of the analyte deposited merely on the Top side increases from *n*=1.0 to *n*=1.5, the corresponding frequency shifts of QBIC I are 18, 29, 48, 65 and 86 GHz, respectively, while QBIC II does not exhibit a significant frequency shift. Fig. S4C shows that as the refractive index of the analyte deposited on the Rear side increases, the corresponding frequency shifts of QBIC II are 34, 67, 95, 129 and 165 GHz, respectively, with no frequency shift in QBIC I. The frequency shifts obtained from the simulations suggest that both the Top and Rear sides of our designed metasurface are capable of performing independent sensing functions. To quantify the dual-sided sensing performance of the metasurface, the frequency shifts of the dual-sided QBIC with varying *n* are extracted. The sensitivity(S), as a widely used sensing indicator, is defined as , where Δ*f* represents the frequency shift and Δ*n* is the change in the refractive index. The calculated sensitivities of QBIC I and QBIC II are 169 GHz/RIU and 325 GHz/RIU, respectively, which are displayed in Fig. S4B and Fig. S4D.

**Fig. S4.** The dual-sided sensing performance analysis in simulation. (A) The spectral changes corresponding to the simulation analysis of the material deposited on the Top sides. (B) The Top side’s sensitivity of the metasurface. (C) The spectral changes corresponding to the simulation analysis of the material deposited on the Rear sides. (D) The Rear side’s sensitivity of the metasurface.

- **Supplementary Note 5**

In addition, the metal metasurface can fulfill the requirement for repeated use, thereby substantially enhancing the cost-effectiveness of the detection process. We conducted multiple performance measurements of the metasurface following repeated experimental trials. We performed THz response measurements on the thoroughly cleaned metasurface following the experiment and the metasurface after multiple functionalization, as shown in the Fig.S5. Fig.S5A presents the THz spectral response of the unprocessed bare metasurface and the bare metasurface after 10 detections. The THz transmission spectra of the metasurface display a high degree of concordance, with only negligible frequency shifts detected. Similarly, we measured the THz transmission spectra of the matesurface following the initial functionalization and after 10 functionalization detections in Fig.S5B, which still remain highly consistent. These results indicate that our metasurface exhibits robust sensing performance even after repeated experiments. The reusable nature of our sensing strategy underscores its cost-effectiveness.


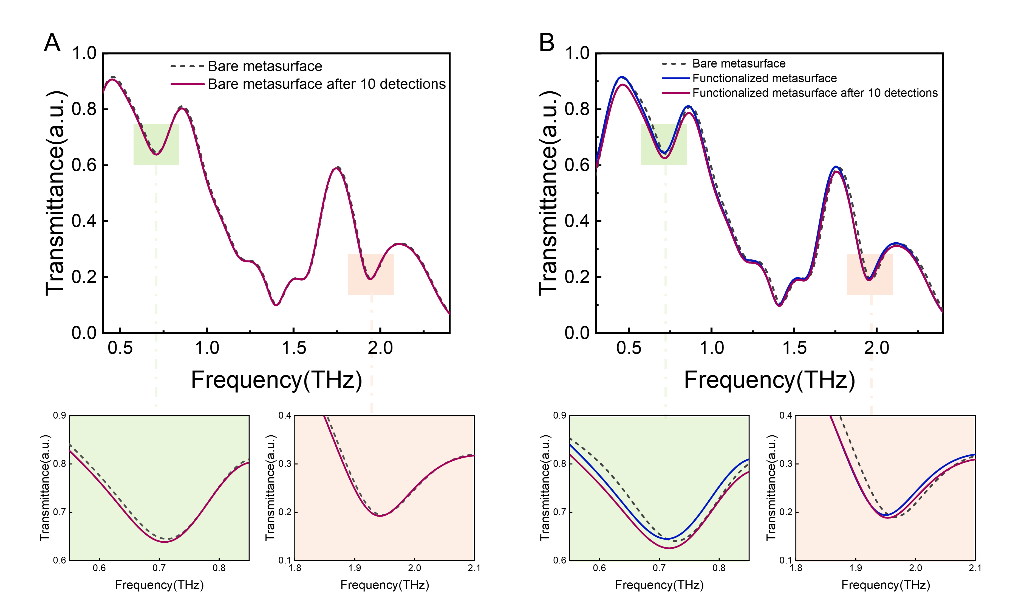


**Fig.S5.** THz spectral response of metasurfaces after multiple experiments. (A) The comparison spectrum of the bare metasurface after 10 detections with the initial bare metasurface. (B) The comparison spectrum of the functionalized metasurface after 10 detections with the initial functionalized metasurface.

- **Supplementary Note 6**

To further validate the enhancement effect of AuNPs on THz detection within our sensing strategy, we performed THz spectroscopic characterization on MGC-803 Exos that were not conjugated with AuNPs. The results are presented in Fig.S6. It is evident that no significant frequency shift was observed at Exos concentrations of 1×104 and 1×105 particles/ml; however, a detectable frequency shift signal became apparent when the concentration reached 1×106 particles/ml. This indicates that the presence of AuNPs significantly amplifies the THz detection signal, thereby improving the minimum detectable concentration of Exos in our sensing strategy by two orders of magnitude.
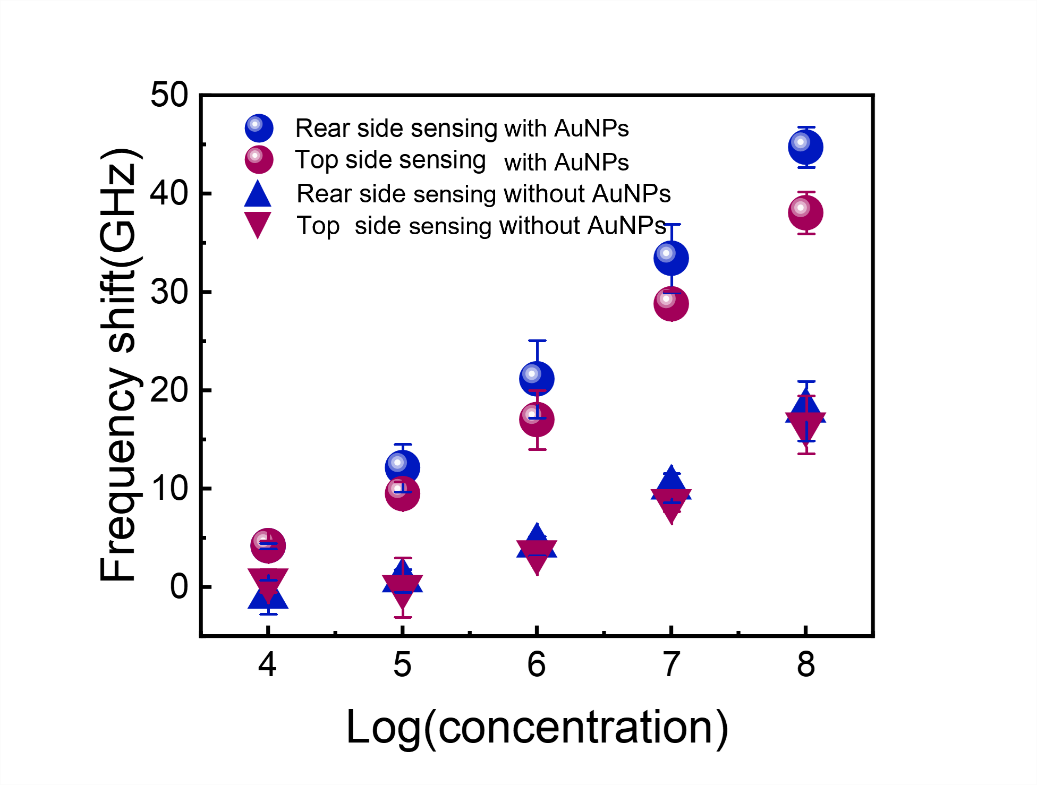


Fig.S6. Dual-sided sensing for the detection of MGC-803 Exos of different concentrations (1×104 -1×108 particles/ml) with/without AuNPs.

- **Supplementary Note 7**

To further test the biosensor's ability to detect different GC-subtypes Exos, we tested Exos derived from GES-1 and SGC-7901 sources with a concentration range from 1×104 to 1×108 particles/ml. Fig. S7A shows the transmission spectrum of the dual-sensing biosensor to the different concentrations of Exos from GES-1. When the concentration of Exos varies from 1×104 to 1×108 particles/ml, the corresponding frequency shifts of QBIC I and QBIC II are 0, 4, 3, 0, 4 GHz and 0, 4, 3, 3, 7 GHz, respectively. Each concentration of Exos was tested three times. Fig. S7C demonstrates the relationship between frequency shift and concentration corresponding to the dual sides. As the concentration of GES-1 Exos increases, neither QBIC shows significant frequency shift. These findings suggest that GES-1 Exos are virtually devoid of CD97 and HMGB1. Fig.S7B shows the transmission spectra of the metasurface corresponding to SGC-7901 Exos. The corresponding frequency shifts of QBIC I and QBIC II are 3, 2, 5, 8, 10 GHz and 0, 6, 10, 17, 26 GHz, respectively. Fig. S7D displays the relationship between frequency shift and concentration corresponding to the dual sides. As the concentration of SGC-7901 Exos increases, QBIC I shows a weak frequency shift trend while QBIC II displays a relatively obvious frequency shift trend. The result reveal that Exos derived from SGC-7901 cells exhibit low levels of CD97 and comparatively high concentrations of HMGB1.

**Fig. S7.** The dual-sensing performance analysis in detecting Exos derived from GES-1 and SGC-7901. (A) The typical transmission spectra depending on GES-1 Exos concentration from 1×104 to 1×108 particles/ml. (B) The typical transmission spectra depending on SGC-7901 Exos concentration from 1×104 to 1×108 particles/ml. The yellow line delineates the trend of frequency shifts, while the gray line indicates no significant frequency shift. (C) The relationship between frequency shift and concentration of GES-1 Exos corresponding to the dual sides. (D) The relationship between frequency shift and concentration of SGC-7901 Exos corresponding to the dual sides.

- **Supplementary Note 8**

To further evaluate the clinical application potential of our sensing strategy, we analyzed Exos derived from 7 patients with GC and 3 normal gastric mucosa specimens. Detailed patient information is provided in Table 1. (This study was approved by the Research Ethics Committee of the Chinese People's Liberation Army General Hospital and was conducted in strict accordance with the principles of the Declaration of Helsinki.)

**Table 1**. Patient information

| **Number** | **Tumor** | **Gender** | **Age** | **cTNM** | **Stage** | **Exos**  **Concentration**  **(particles/ml)** | **CD97** | **HMGB1** |
| --- | --- | --- | --- | --- | --- | --- | --- | --- |
| **N1** | normal | M | 49 | ***** | ***** | 2.85×1010 | low | low |
| **N2** | normal | F | 51 | ***** | ***** | 3.05×1010 | low | low |
| **N3** | normal | F | 37 | ***** | ***** | 2.07×1010 | low | low |
| **P1** | gastric cancer | F | 70 | T3N0M0 | IIA | 3.74×1010 | low | high |
| **P2** | gastric cancer | M | 41 | T2N0M0 | I | 2.33×1010 | low | high |
| **P3** | gastric cancer | M | 52 | T2N0M0 | I | 3.17×1010 | low | moderate |
| **P4** | gastric cancer | F | 70 | T3N0M0 | IIA | 2.68×1010 | high | high |
| **P5** | gastric cancer | F | 67 | T4aN2bM1 | IV | 3.59×1010 | high | high |
| **P6** | gastric cancer | F | 52 | T4aN1bM0 | IIIB | 4.55×1010 | high | high |
| **P7** | gastric cancer | M | 64 | T4aN2aM0 | IIIC | 2.32×1010 | high | high |

The images of these specimens are shown in Fig.S8. Exos were isolated from each tissue sample via density gradient centrifugation and size-exclusion chromatography.

**
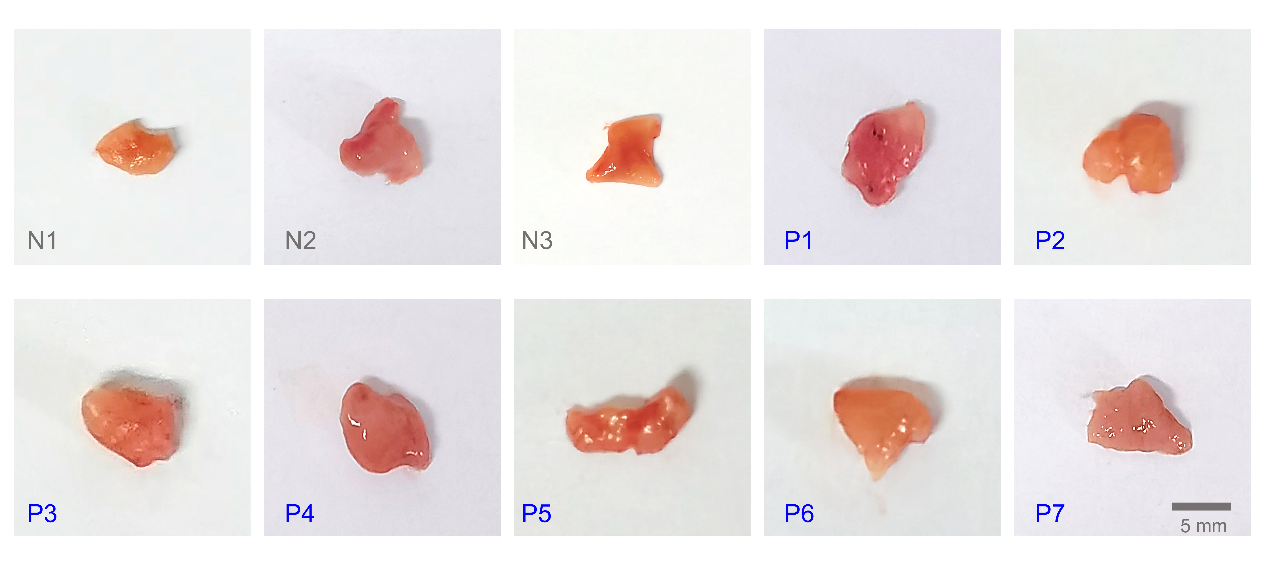
**

**Fig.S8.** Tissue images from 3 normal control patients and 7 GC patients.

The expression levels of CD97 and HMGB1 within these Exos were subsequently assessed using Western blotting, as depicted in Fig.S9. As displayed in Fig.S9A, CD97 and HMGB1 were undetectable in all 3 healthy samples (N1, N2, and N3). We conducted a study involving GC patients at various pathological stages. The findings revealed that in early-stage GC cases (P1, P2, P3, and P4, with stage I or IIA, do not require subsequent chemotherapy) with lower invasion depth, the expression levels of CD97 were relatively low, whereas HMGB1 exhibited a moderate increase. In contrast, in patients with advanced GC (P5, P6, and P7), both CD97 and HMGB1 showed significantly elevated expression levels. The complete WB diagram is shown in Fig.S9B and Fig.S9C.


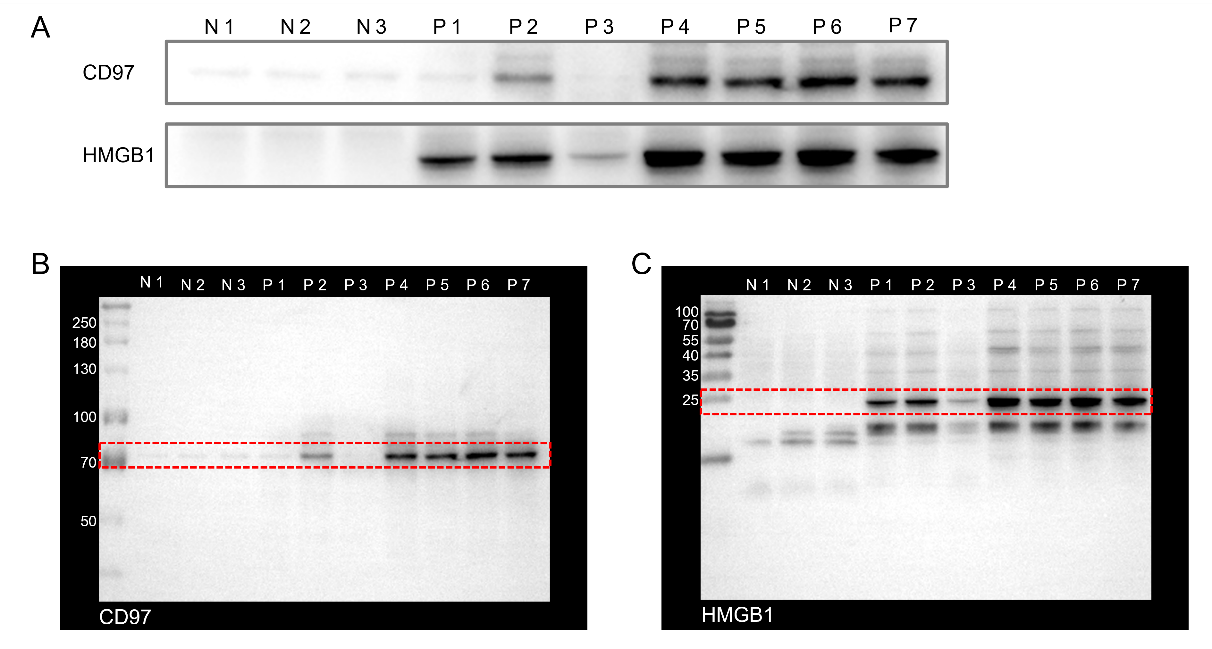


**Fig.S9.** The expression levels of CD97 and HMGB1 in Exos derived from three normal individuals and seven GC tissues. (A) Expression levels of CD97 and HMGB1 in ten Exos samples. (B), (C) The complete Western blot image of CD97 and HMGB1.

Exos isolated from each sample were formulated into solutions at a concentration of 1×108 particles/mL in PBS and subsequently analyzed by our sensing platform. The results of dual QBIC frequency shifts are presented in Fig.S10A. The frequency shifts of QBIC I and QBIC II are generally consistent with the levels of exosomal membrane proteins observed in the Western blot analysis (Fig.S9A), which indicates that our sensing strategy can accurately detect two exosomal membrane proteins simultaneously, and has great potential in personalized treatment and prognosis assessment for patients. We conducted a comparative analysis of QBIC I and QBIC II across three groups (N1-3, P1-4, and P5-7) based on the tumor stage and whether chemotherapy is needed. The frequency shift results for each group are presented in Fig.S10B. Our sensing platform is capable of simultaneously detecting two exosomal membrane proteins, thereby not only effectively distinguishing Exos from normal tissues and GC but also further analyzing inter-individual variations in Exos content, demonstrating possible potential for predicting patient pathological stages and assessing prognosis. Compared to Exos obtained from cell lines cultures, this platform exhibits a lower frequency shift when detecting clinical samples, which may be attributed to the complexity of tumor tissue components (mesenchymal cells, endothelial cells, and immune cells). Given that both CD97 and HMGB1 promote tumor infiltration and metastasis, and considering the current sample size, our platform demonstrates preliminary capability in distinguishing different tumor pathological types, thus providing guidance for targeted therapy and prognosis assessment of patients.

**Fig.S10.** Clinical detection of Exos using dual-sided sensing platform. (A) The frequency shifts of QBIC I and QBIC II in three healthy controls and seven GC patients. (B) Comparison of frequency shifts between the healthy group (N1-3) and different pathological stage groups (P1-4, P5-7).

- **Supplementary Note 9**

In this work, aiming to tackle the heterogeneity of exosomal membrane proteins to achieve the differentiation of tumor subtypes, we introduce a dual-sensing THz metasensor detecting two membrane proteins simultaneously in single-step. Compared with previous detection methods (ELISA, Fluorescence, Electrochemical) in Table 2, our work provides a new dual-sided sensing strategy based on THz and metasurfaces. Our dual-sided detection system unequivocally augments the reliability of trace detection capabilities for the reason of dual-sensing and substantiate the biosensor's proficiency in discerning GC-subtypes Exos based on two Exosomal membrane proteins. Compared with previous Thermophoresis that can distinguish Exos subtypes, our work displays an extensive dynamic response range coupled with a satisfied low detection threshold (LOD is reduced by an order of magnitude). To sum up, our work is grounded in an innovative dual-sensing methodology that offers enhanced detection capabilities, showing promising prospects in clinical settings like personalized treatment for individuals with GC.

**Table 2. Comparison of different Exos detection methods.**

| **Ref.** | **Method** | **Detection Principle** | **Target Exos** | **Detection**  **range**  **(particles/ml)** | **Sensitivity**  **(particles/ml)** | **specificity** | **Detection speed** | **Cancer-subtype**  **Exos analysis** |
| --- | --- | --- | --- | --- | --- | --- | --- | --- |
| [41] | ELISA | Absorbance | Breast  cancer cell | 1 × 104 -  1 × 108 | 1.0 × 104 | Specific capture antibody | > 4h | NO |
| [42] | Fluorescence | Fluorescence intensity | Gastric  cancer cell | 1 × 105 -  1 × 109 | 4.2 × 104 | an aptamer to target specific  exosomes | >8h | NO |
| [43] | Electrochemical | Current  intensity | Gastric  cancer cell | 4.8 × 103 -  4.8 × 106 | 9.5 × 102 | aptasensor and antibody | >2h 10min | NO |
| [44] | Thermophoresis | DNA  computation | Breast  cancer cell | 2 × 106 -  2 × 109 | 2.8 × 105 | the aptamer-based logic gate | >3.5h | YES |
| This work | THz metasurface biosensor | Dual-sensing mode | Gastric  cancer cell | 1 × 104 -  1 × 108 | 1.0 × 104 | Antibody- membrane protein affinity | 1.5h | YES |
