# Supplementary figures and images for: Integrated Single-Step Terahertz Metasensing for Simultaneous Detection Based on Exosomal Membrane Proteins Enables Pathological Typing of Gastric Cancer
Source: Research (Wash D C). 2025 Mar 10;8:0625. doi: 10.34133/research.0625 (PMC11891340; doi:10.34133/research.0625)

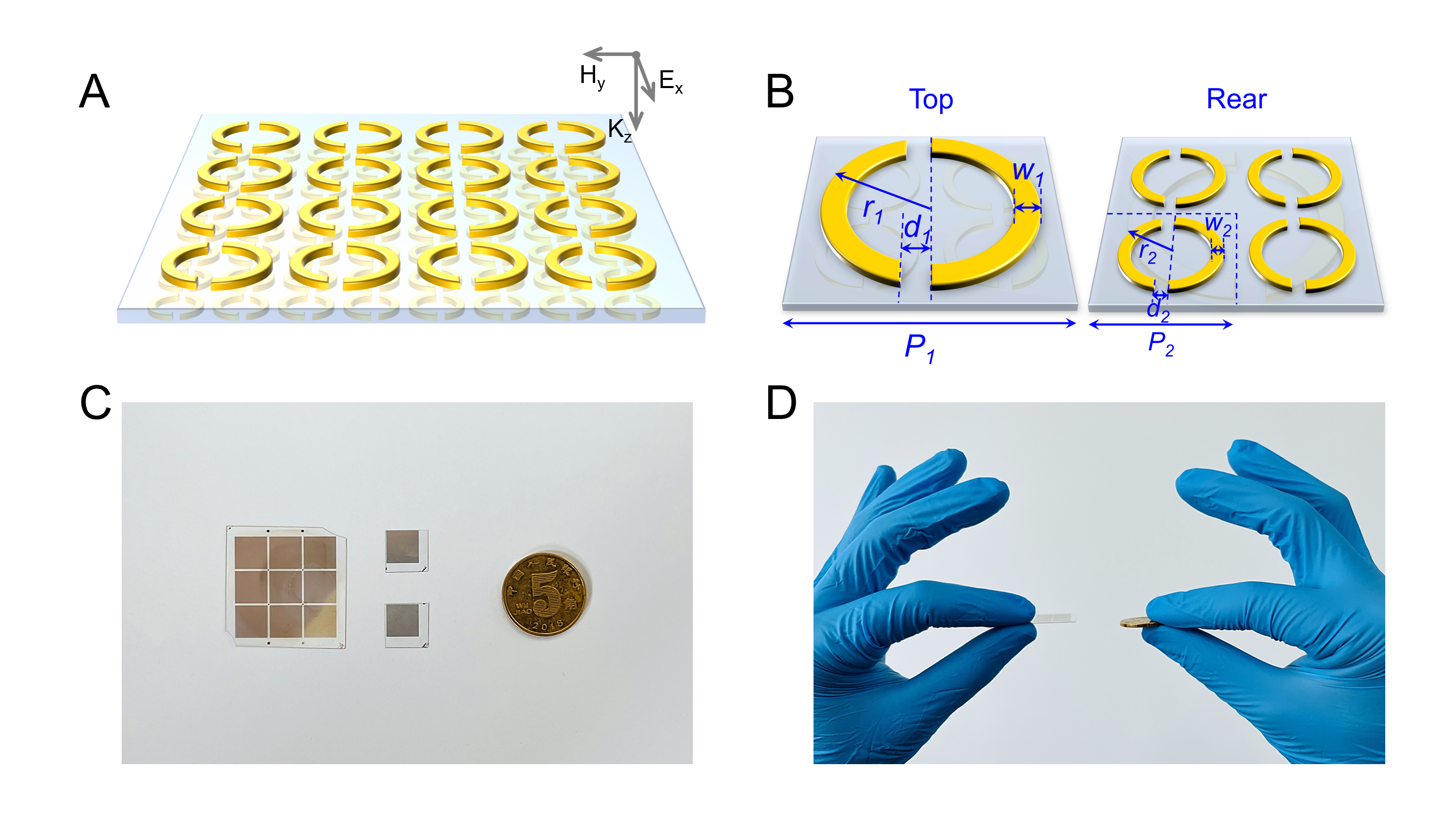

Supplement: Supplementary 1 — Notes S1 to S9 Figs. S1 to S10 Tables S1 and S2 [file research.0625.f1.zip › Fig.S1.tif]

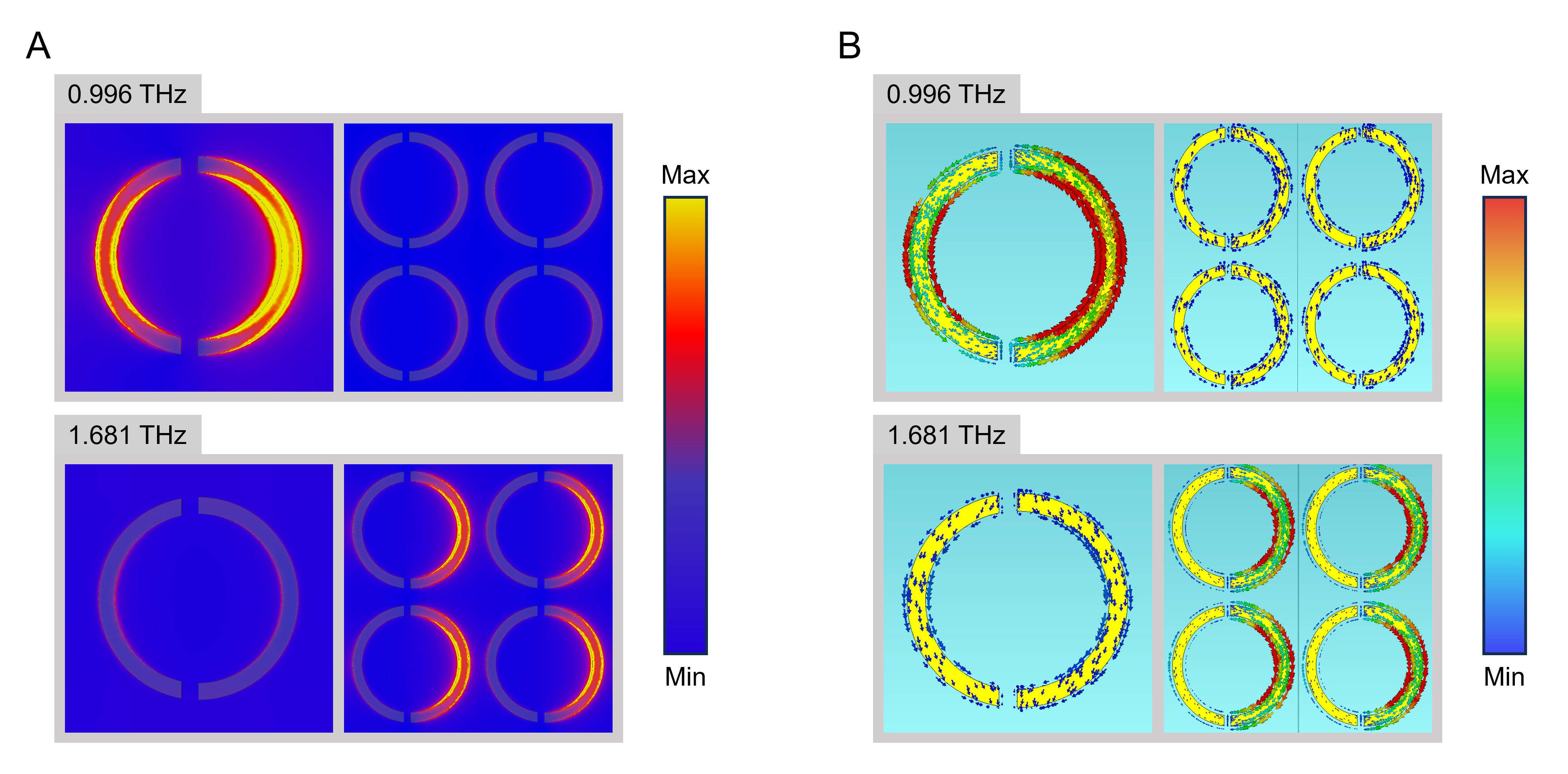

Supplement: Supplementary 1 — Notes S1 to S9 Figs. S1 to S10 Tables S1 and S2 [file research.0625.f1.zip › Fig.S3.tif]

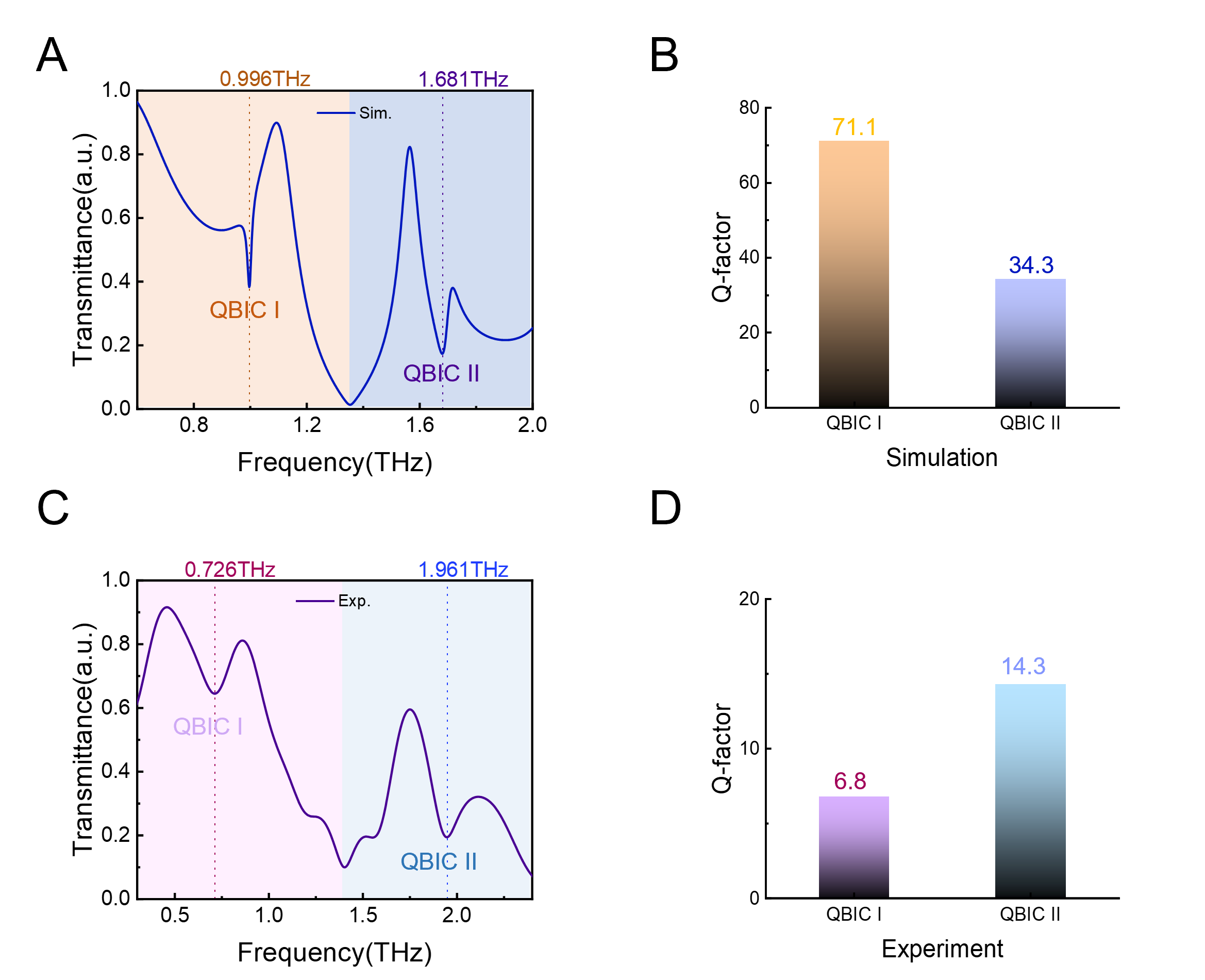

Supplement: Supplementary 1 — Notes S1 to S9 Figs. S1 to S10 Tables S1 and S2 [file research.0625.f1.zip › Fig.S2.tif]

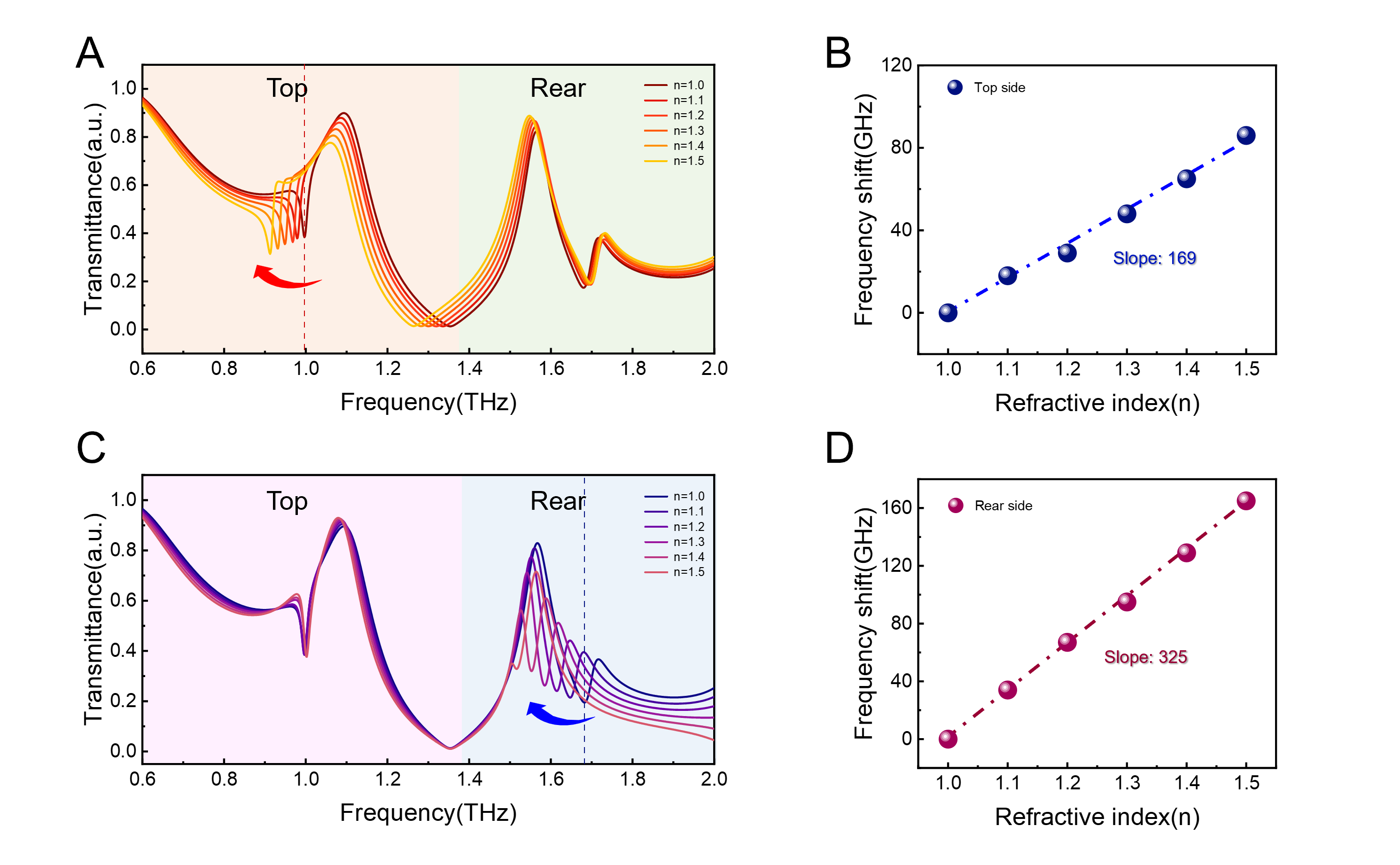

Supplement: Supplementary 1 — Notes S1 to S9 Figs. S1 to S10 Tables S1 and S2 [file research.0625.f1.zip › Fig.S4.tif]

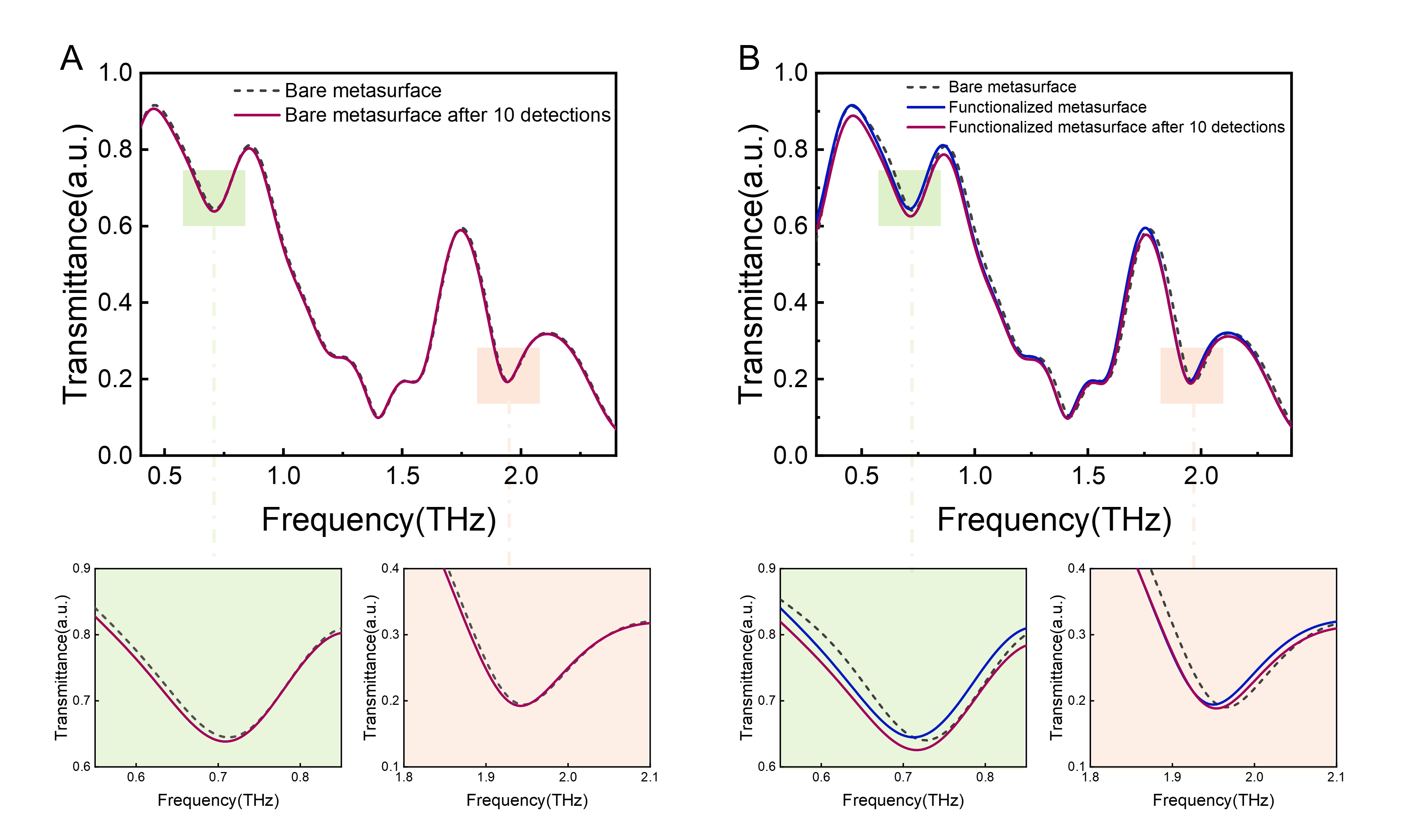

Supplement: Supplementary 1 — Notes S1 to S9 Figs. S1 to S10 Tables S1 and S2 [file research.0625.f1.zip › Fig.S5.tif]

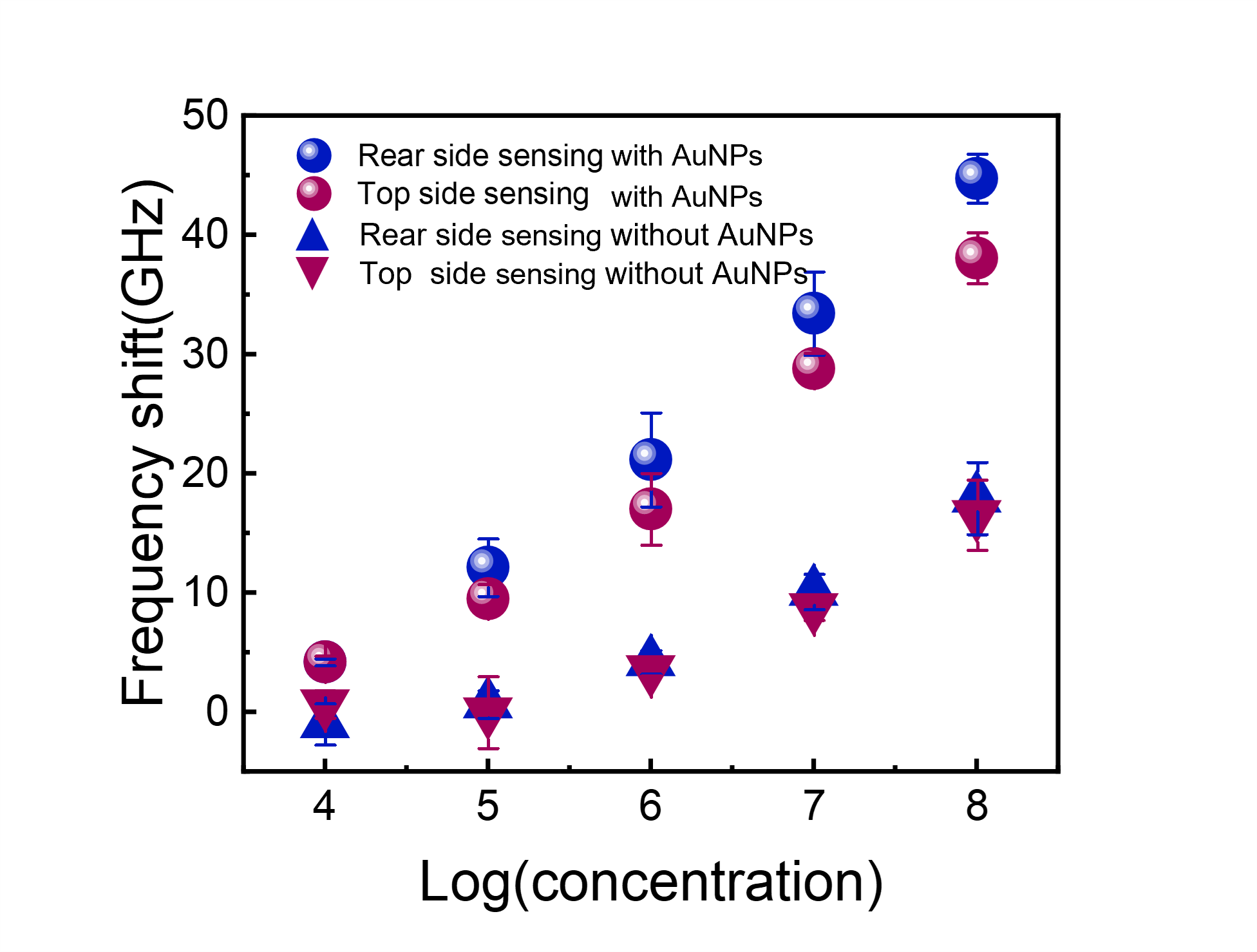

Supplement: Supplementary 1 — Notes S1 to S9 Figs. S1 to S10 Tables S1 and S2 [file research.0625.f1.zip › Fig.S6.tif]

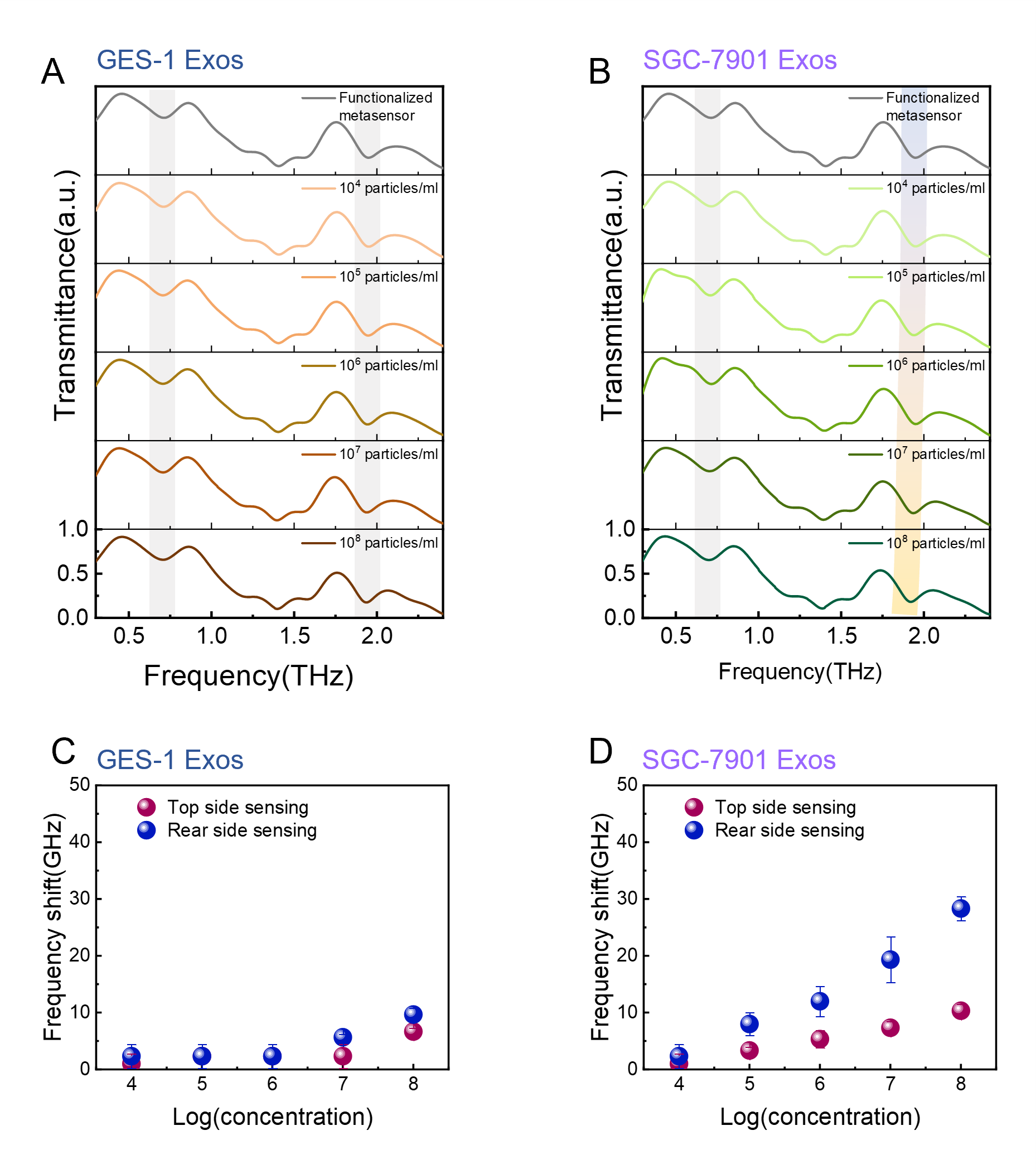

Supplement: Supplementary 1 — Notes S1 to S9 Figs. S1 to S10 Tables S1 and S2 [file research.0625.f1.zip › Fig.S7.tif]

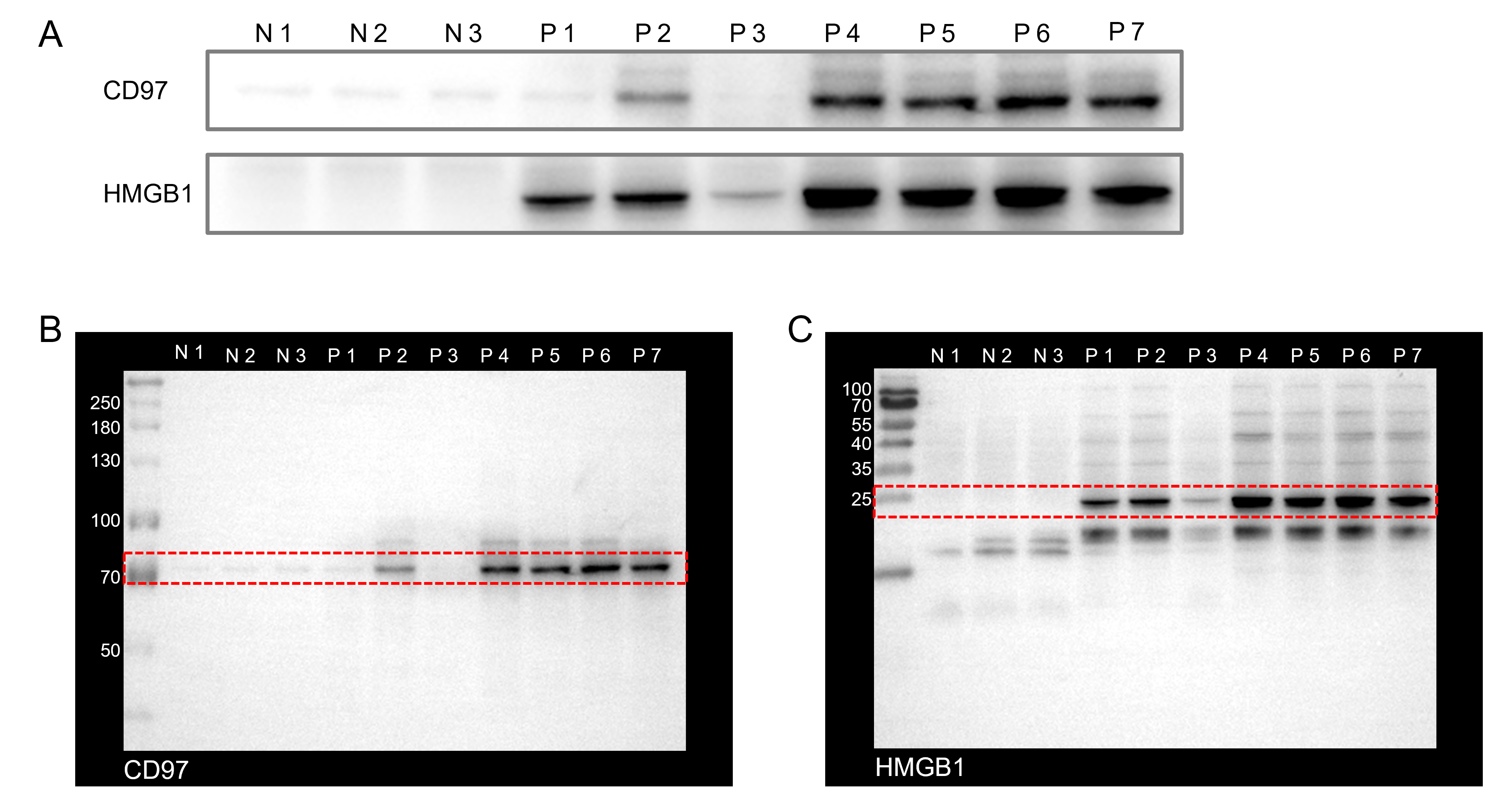

Supplement: Supplementary 1 — Notes S1 to S9 Figs. S1 to S10 Tables S1 and S2 [file research.0625.f1.zip › Fig.S9.tif]

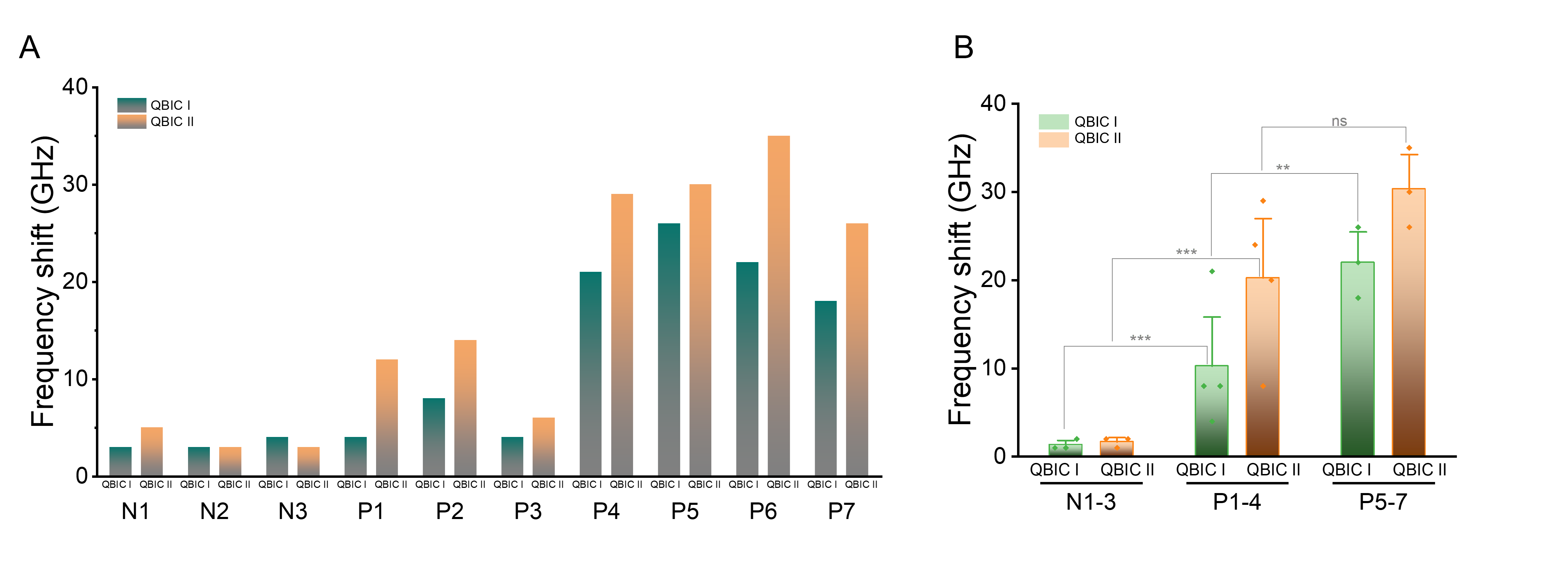

Supplement: Supplementary 1 — Notes S1 to S9 Figs. S1 to S10 Tables S1 and S2 [file research.0625.f1.zip › Fig.S10.tif]

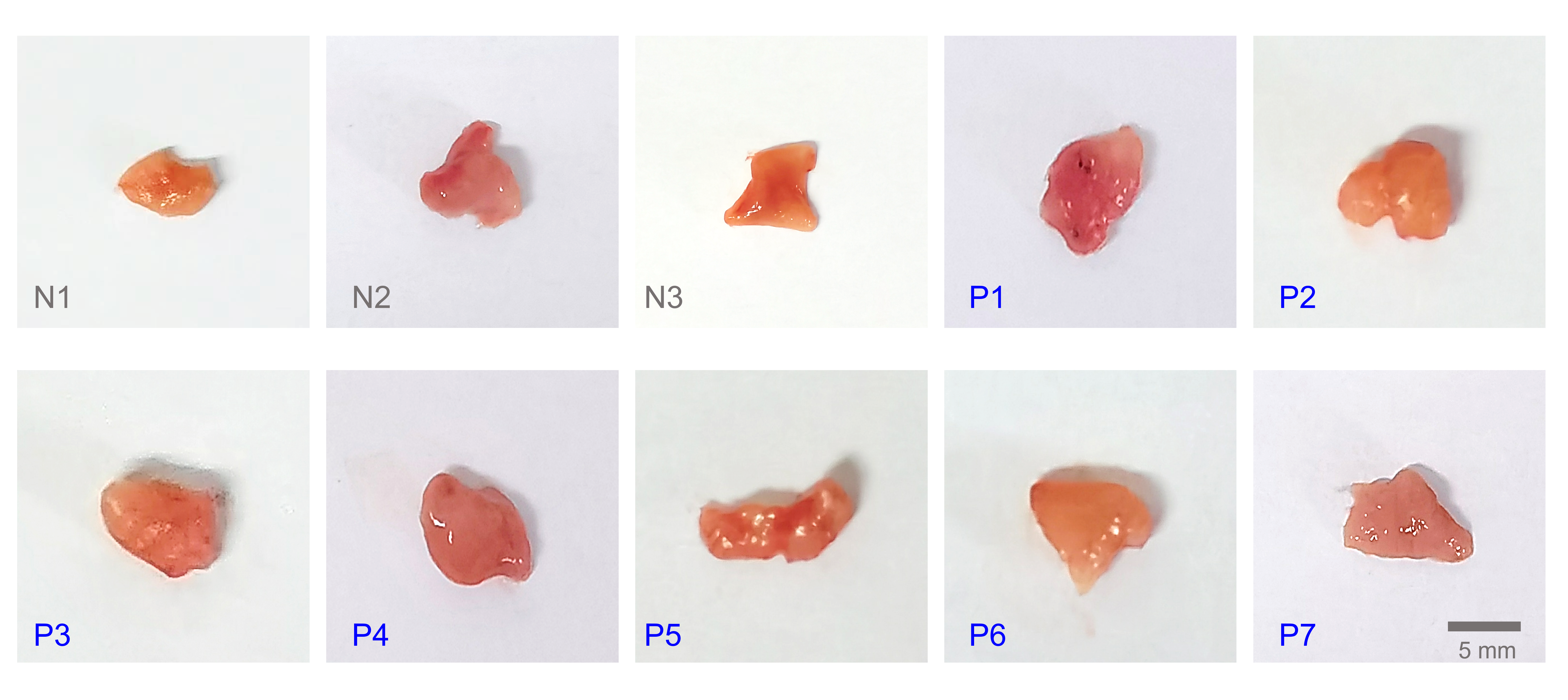

Supplement: Supplementary 1 — Notes S1 to S9 Figs. S1 to S10 Tables S1 and S2 [file research.0625.f1.zip › Fig.S8.tif]
