# Supplementary figures and images for: Erratum to “Integrated Single-Step Terahertz Metasensing for Simultaneous Detection Based on Exosomal Membrane Proteins Enables Pathological Typing of Gastric Cancer”
Source: Research (Wash D C). 2026 Apr 20;9:1247. doi: 10.34133/research.1247 (PMC13093893; doi:10.34133/research.1247)

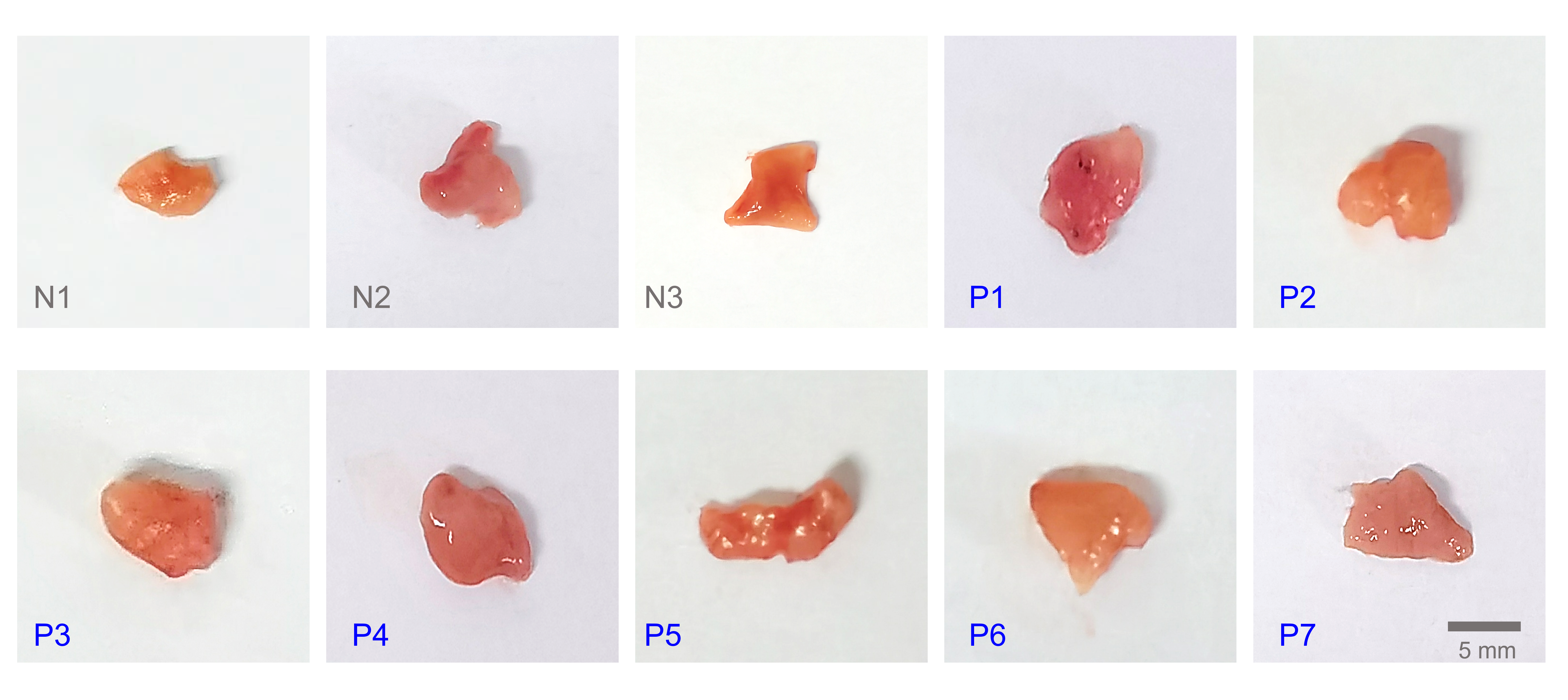

Supplement: Supplementary 1 — Supplementary Notes S1 to S9 Figs. S1 to S10 Tables S1 and S2 [file research.1247.f1.zip › revised-S8.tif]
